# *Kalanchoe crenata* Andrews (Haw.) Improves Losartan’s Antihypertensive Activity

**DOI:** 10.3390/molecules29246010

**Published:** 2024-12-20

**Authors:** Pedro de Padua G. Amatto, Juliana da Silva Coppede, Carla Renata Kitanishi, Giovana Graça Braga, Thaysa Carvalho de Faria, Elen Rizzi, Suzelei de Castro França, Fernanda Basso, Adriana Aparecida Lopes, Fábio Carmona, Silvia Helena Taleb Contini, Ana Maria Soares Pereira

**Affiliations:** 1 Department of Biotechnology of Medicinal Plants, University of Ribeirão Preto, Ribeirão Preto 14096-900, Brazil; pedro.goulart@sou.unaerp.edu.br (P.d.P.G.A.); jcoppede@unaerp.br (J.d.S.C.); ckitanishi@unaerp.br (C.R.K.); gi07braga@usp.br (G.G.B.); thaysa.tinki@hotmail.com (T.C.d.F.); elen_rizzi@yahoo.com.br (E.R.); sfranca@unaerp.br (S.d.C.F.); alopes@unaerp.br (A.A.L.); scontini@unaerp.br (S.H.T.C.); 2 School of Dentistry, São Paulo State University Júlio de Mesquita Filho, Araraquara 14800-060, Brazil; f.basso@unesp.br; 3 Ribeirão Preto Medical School, University of São Paulo, Ribeirão Preto 14049-900, Brazil; carmona@usp.br; 4 Botanical Garden of Medicinal Plants Ordem e Progresso, Jardinopólis 14680-000, Brazil

**Keywords:** patuletins, hypertension, medicinal plant, systolic blood pressure, murine model

## Abstract

Background: Cardiovascular diseases constitute one of the leading causes of morbidity and mortality worldwide. Herbal medicines represent viable alternatives to the synthetic drugs currently employed in the control of hypertension. This study aimed to isolate and identify the chemical markers of *Kalanchoe crenata* and to investigate the antihypertensive and anti-matrix metalloproteinase (MMP2) activities of an aqueous extract of the leaves. Methods: The main constituents of the aqueous extract of *K. crenata* were separated by ultra-performance liquid chromatography–mass spectrometry, and their presence was identified by NMR spectroscopy. Renovascular hypertension was induced in male Wistar rats using the two-kidney one-clip method (HTN groups), while control animals (Sham groups) were submitted to Sham surgery. Six groups of 10 animals each were treated daily for eight weeks as follows: Sham 1 (carrier), Sham 2 (*K. crenata* extract), HTN.1 (carrier), HTN.2 (*K. crenata* extract), HTN 3 (losartan), and HTN 4 (*K. crenata* extract with losartan). Results: The main compounds of the extract were patuletin 3-*O*-(4″-*O*-acetyl-α-L-rhamnopyranosyl)-7-*O*-(3‴-*O*-acetyl-α-L-rhamnopyranoside) (**1**), patuletin 3-*O*-α-L-rhamnopyranosyl-7-*O*-L-rhamnopyranoside (**2**), and *trans*-caffeoyl-malic acid (**3**), with compounds **1** and **2** being chemical markers of the species. Significant reductions (*p* < 0.05) in systolic blood pressure and MMP2 (72kDa isoform) activity were observed in the HTN 4 group. Conclusions: The association of *K. crenata* extract and losartan presented in vivo effects against hypertension.

## 1. Introduction

Cardiovascular diseases are the cause of approximately 32% of all deaths worldwide [1,2]. Hypertension, which is characterized by systolic blood pressure (SBP) ≥ 140 mmHg and diastolic blood pressure ≥ 90 mmHg, is alone responsible for more than 7.5 million deaths annually [3]. Diverse medications, including diuretics, calcium channel blockers, angiotensin-converting enzyme (ACE) inhibitors, angiotensin II receptor blockers, beta blockers, and vasodilators, are currently available to control hypertension [4]. Nevertheless, some 30% of patients do not respond to these drugs and remain vulnerable to morbidities caused by the untreated disease [5,6].

Increases in extracellular matrix metalloproteinases (MMPs), an important family of zinc-dependent endopeptidases involved in cell repair and the remodeling of cardiac and vascular tissues, are implicated in the pathophysiology and progression of hypertension [7]. Thus, compounds or extracts of plant origin that reduce or modulate MMPs may represent viable alternatives in the treatment of hypertension, especially when they possess vasodilatory activity through the inhibition of ACE or blocking of calcium channels. In this sense, several pre-clinical and clinical trials have demonstrated that some herbal medicines with known mechanisms of action are effective in controlling hypertension [8,9,10,11].

Among the many species of medicinal plants that possess verifiable cardiovascular properties, appreciable research attention has focused on members of the genus *Kalanchoe* (Crassulaceae). For example, aqueous extracts of *Kalanchoe pinnata* (Lam.) Pers. reportedly exhibit an antihypertensive activity comparable with that of spironolactone, reduce the levels of creatine kinase and troponin, and prevent heart damage, all of which are associated with the antioxidant activity of the species [12].

Another species of interest is *Kalanchoe crenata* Andrews (Haw.) (basionym = *Kalanchoe integra* var. *crenata* (Andrews) Cufod. or synonym = *Kalanchoe brasiliensis* Cambess.). There are records of its traditional uses in Brazil and African countries for the treatment of depression, smallpox, otitis, cough, asthma, palpitations, hypertension, headaches, convulsions, and general weakness [13,14,15,16]. Furthermore, many studies have demonstrated that extracts of *K. crenata* engender a range of effects on cardiac cells and receptors, including decreased insulin resistance, increased superoxide dismutase and catalase activities, and reduced levels of malondialdehyde, total cholesterol, and triglycerides [17,18].

In an early study, it was reported that an *n*-butanol extract of the leaves of *K. crenata* reduced blood pressure significantly in normotensive rats and suggested that the effect may be related to the blocking of the potassium channel [19]. Moreover, an ethanolic extract of the leaves of *K. crenata* protected Sprague-Dawley rats from doxorubicin-induced cardiotoxicity, possibly by modulating nitric oxide production [20].

The juice of fresh stem and leaves of *K. crenata* contains patuletin acetyl rhamnosides [21], and the aglycone patuletin has recently been reported to reduce acute N(gamma)-nitro-L-arginine methyl ester (L-NAME)-induced hypertension in rat models [22]. These findings suggest that the mechanism of action of the extracts is probably associated with the modulation of nitric oxide release.

Considering the positive effects of *K. crenata* on the pathophysiology of cardiovascular diseases and its traditional use in the mitigation of heart palpitations [16], the aims of the present study were (i) to isolate, identify, and quantify the chemical markers (bioactive principles) in an aqueous extract of *K. crenata*, (ii) to investigate the antihypertensive activity of the aqueous extract, alone and in combination with losartan, and (iii) to evaluate the effects of the extract on the activity of aortic MMP2.

## 2. Results

### 2.1. Structural Identification of Compounds Isolated from K. crenata Leaves

An aqueous extract of the fresh leaves of *K. crenata* was partitioned sequentially against organic solvents of increasing polarity, and the *n*-butanol fraction was separated by preparative HPLC to yield three pure samples, namely Kc3-83, Kc63, and Kcb.

**Figure 1:**
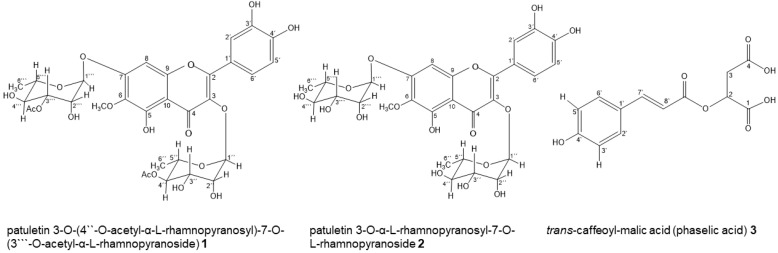
Compounds isolated from the aqueous extract of *Kalanchoe crenata* leaves are as follows: **1**—patuletin 3-*O*-(4″-*O*-acetyl-α-L-rhamnopyranosyl)-7-*O*-(3‴-*O*-acetyl-α-L-rhamnopyranoside); **2**—patuletin 3-*O*-α-L-rhamnopyranosyl-7-*O*-L-rhamnopyranoside; and **3**—*trans*-caffeoyl-malic acid or phaselic acid.

Comparison of the ^1^H, ^13^C, HSQC, HMBC, and COSY NMR spectra obtained for sample Kc3-83 with values described in the literature [21,23] allowed the identification of patuletin 3-*O*-(4″-*O*-acetyl-α-L-rhamnopyranosyl)-7-*O*-(3‴-*O*-acetyl-α-L-rhamnopyranoside) (compound **1**; Figure 1). The ^1^H NMR spectrum (Supplementary File, Figure S3) showed the presence of characteristic signals corresponding to aromatic hydrogens between δ 6.65 and δ 7.39 and glycosidic hydrogens between δ 0.80 and δ 5.60, indicating that compound **1** was a glycosylated flavonoid. In the aglycone, the singlet observed at δ 6.65 was attributed to H-8, suggesting a penta-substituted A ring, while the doublet at δ 6.94 (*J* = 8 Hz), the doublet of doublets at δ 7.33 (*J* = 8.0, 2.0 Hz), and the doublet at δ 7.39 (*J* = 2Hz) were attributed, respectively, to H-5′, H-6′, and H-2′ in the B ring. The singlet at δ 3.93 correlated with three hydrogens referring to the methoxyl group (-OCH_3_) at C-6, which characterized the aglycone as the flavonol patuletin (6-methoxyquercetin or quecetagetin 6-methyl ether) (Supplementary File, Table S1).

Regarding the signals corresponding to the glycosidic components of compound **1**, the two broad singlets at δ 5.60 and δ 5.52 may be attributed to anomeric hydrogens, while the doublets at δ 0.80 (*J* = 6.0 Hz) and δ 1.29 (*J* = 6 Hz) are typical of methyl hydrogens (-CH_3_), indicating the presence of two rhamnose units linked to the flavonoid moiety. According to Harborne [24], when rhamnose is linked to the C3 of the flavonoid ring, the chemical shifts of the methyl and anomeric hydrogens are, respectively, in the regions δ 0.72–δ 0.86 and δ 4.96–δ 5.36. Conversely, when rhamnose is linked to the C7 of the aglycone, the chemical shifts of the methyl and anomeric hydrogens are, respectively, in the regions δ 1.04–δ 1.21 and δ 5.22–δ 5.75.

The ^13^ C NMR spectrum of compound **1** (Supplementary File, Figure S4) showed signals at δ 17.61 and δ 18.09, which are characteristic of methyl carbons (C6″ and C6‴) of the two rhamnose units, and at δ 102.61 and δ 100.23, corresponding to the anomeric carbons C1″ and C1‴, respectively. Furthermore, the signals at δ 69.70 and δ 71.71, attributed to C5″ and C5‴, respectively, indicated the presence of two α-rhamnose units [25]. Additionally, the two signals at δ172.67 and δ172.56 could be attributed to the carbonyl carbons of the acetyl groups linked to C4″ and C3‴ of the rhamnose units. The two-dimensional HSQC spectrum of compound **1** (Supplementary File, Figure S5) confirmed the predicted correlations between the signals of the hydrogens and the carbons in the molecule.

The HMBC spectrum of compound **1** (Supplementary File, Figure S6) showed ^3^*J* couplings (^1^H-^13^C) of the anomeric H-1″ (at δ 5.52) in rhamnose with C3 (at 135.78 ppm) of the aglycone patuletin, and of the anomeric H-1‴ (at δ 5.60) in rhamnose unit with C7 (at δ 156.23) of the aglycone A ring, indicating the two positions of glycosylation. The HMBC spectrum further revealed that (i) the rhamnose unit linked to C3 was acetylated at the C4″ position since H4″ at δ 4.86 was correlated with the carbonyl carbon at δ 172.67, and (ii) the second rhamnose unit linked to C7 was acetylated at the C3‴ position since H-3‴ at δ 5.19 was correlated with the carbonyl carbon at δ 172.56. The correlations observed in the two-dimensional ^1^H-^1^H COSY spectrum (Supplementary File, Figure S7) facilitated the characterization of the hydrogen sequences of the rhamnose units.

Comparison of the NMR spectra obtained for sample Kc63 with values described in the literature [21] allowed the identification of patuletin 3-*O*-α-L-rhamnopyranosyl-7-*O*-L-rhamnopyranoside (compound **2**; Figure 1). The ^1^H and ^13^C NMR spectra of compound **2** (Supplementary File, Figures S8 and S9, Table S2) were similar to those of compound **1** except for the absence of the hydrogen and carbon signals of the acetyl groups. The signals observed in the HSQC, HMBC, and 1H-1H COSY spectra (Supplementary File, Figures S10–S12) facilitated the identification of compound **2**.

Comparison of the NMR spectra obtained for sample Kcb with values described in the literature [26] allowed the identification of *trans*-caffeoyl-malic acid (phaselic acid) (compound **3**; Figure 1). The ^1^H NMR spectrum of compound **3** (Supplementary File, Figure S13) showed signals between δ 6.31 and δ 7.59 that are characteristic of aromatic hydrogens present in a caffeoyl unit. The ^13^C NMR spectrum (Supplementary File, Figure S14) showed 13 signals, including those at δ 147.91 and δ 114.23 attributed to carbons C7′ and C8′, respectively, and at δ 168.13 attributed to carbon C9′, all present in the caffeoyl unit (Supplementary File, Table S3). Malic acid was identified by the typical signals of H2 at δ 5.47 (dd; *J* = 8.0, 4.0 Hz), H3a at δ 2.97 (dd; *J* = 16.0, 4.0 Hz), and H3b at δ 2.88 (dd; *J* = 16.0, 4.0 Hz). The HSQC spectrum (Supplementary File, Figure S15) confirmed the correlation between hydrogen and carbon signals (Supplementary File, Table S3). In contrast, the two-dimensional HMBC spectrum (Supplementary File, Figure S16) showed the ^3^*J* (^1^H-^13^C) couplings of H2 of malic acid at δ 5.42 with the C9′ of the carbonyl carbon at δ 168.13, demonstrating that malic acid is linked to the caffeoyl group. The correlation between H3b at δ 2.88 and H2 at δ 5.47, observed in the ^1^H-^1^H COSY spectrum, confirmed the presence of malic acid in the molecule (Supplementary File, Figure S17).

Quantitative analysis of the aqueous extract from *K. crenata* by HPLC with diode array detection (Supplementary File, Figure S18) revealed the presence of patuletin 3-*O*-(4″-*O*-acetyl-α-L-rhamnopyranosyl)-7-*O*-(3‴-*O*-acetyl-α-L-rhamnopyranoside) (**1**) and patuletin 3-*O*-α-L-rhamnopyranosyl-7-*O*-L-rhamnopyranoside (**2**) at concentrations of 167 and 131.77 μg/g of fresh leaves, respectively.

### 2.2. Effects of the Aqueous Extract from K. crenata Leaves

**Figure 2:**
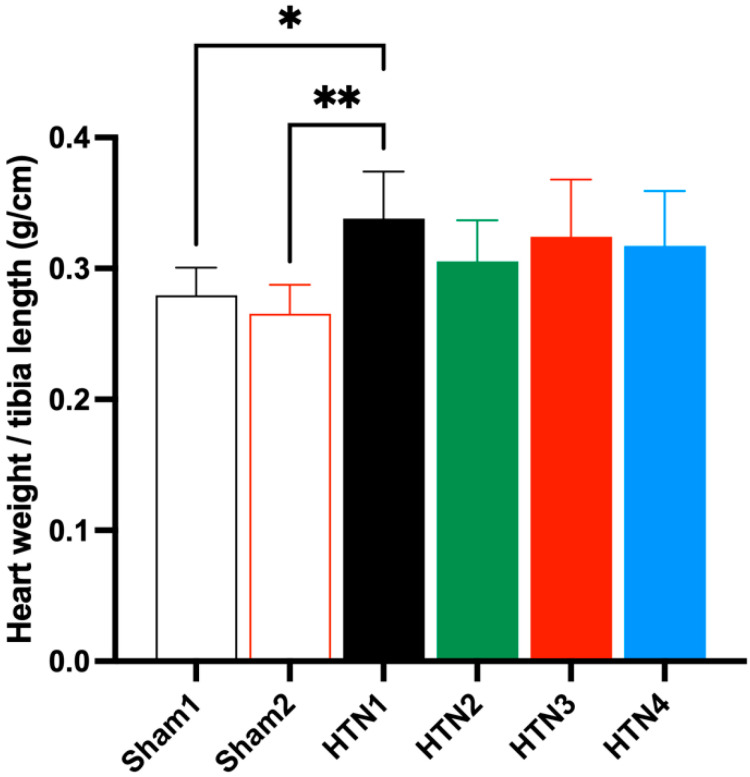
Cardiac hypertrophy indices of normotensive and hypertensive rats determined as the ratio of heart weight (g) to tibia length (cm). Normotensive rats were submitted to Sham surgery and treated with the carrier alone (Sham 1) or with 300 mg/kg *Kalanchoe crenata* extract (Sham 2). Hypertensive rats were submitted to the two-kidney one-clip (2K1C) procedure and treated with the carrier alone (HTN 1), with 300 mg/kg *K. crenata* extract (HTN 2), with 10 mg losartan (HTN 3), or with 300 mg/kg *K. crenata* extract and 10 mg losartan (HTN 4). Significant differences in relation to HTN1 are indicated by asterisks as follows: *p* < 0.05 (*) and *p* < 0.01 (**).

**Figure 3:**
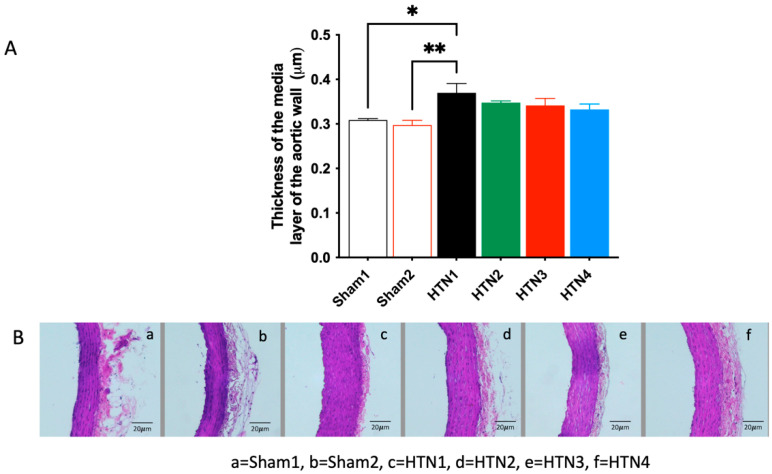
Thickness of the middle layer of the aortic wall of normotensive and hypertensive rats as shown by the following: (**A**) measurements taken from images obtained using an optical microscope and processed with the help of ImageJ 1.46r software; and (**B**) sections stained with hematoxylin and eosin. Normotensive rats were submitted to Sham surgery and treated with the carrier alone (Sham 1) or with 300 mg/kg *Kalanchoe crenata* extract (Sham 2). Hypertensive rats were submitted to the two-kidney one-clip (2K1C) procedure and treated with the carrier alone (HTN 1), with 300 mg/kg *K. crenata* extract (HTN 2), with 10 mg losartan (HTN 3), or with 300 mg/kg *K. crenata* extract and 10 mg losartan (HTN 4). Significant differences in relation to HTN1 are indicated by asterisks as follows: *p* < 0.05 (*) and *p* < 0.01 (**).

Cardiac hypertrophy indices were similar for all animals in the HTN groups regardless of the treatment applied, namely carrier (HTN 1), *K. crenata* extract (HTN 2), losartan (HTN 3), or *K. crenata* extract with losartan (HTN 4) (Figure 2). However, the indices of all HTN animals were significantly higher than those of both Sham 1 animals treated with carrier (*p* < 0.05) and Sham 2 animals treated with *K. crenata* extract (*p* < 0.01). These results imply that the induction of hypertension using the 2K1C model was successful. A similar situation was observed regarding the thickness of the middle layer of the aortic wall, the values of which were similar for all animals in groups HTN 1–4, regardless of the treatment applied, but significantly greater than those of Sham 1 (*p* < 0.05) and Sham 2 (*p* < 0.01) animals (Figure 3).

**Figure 4:**
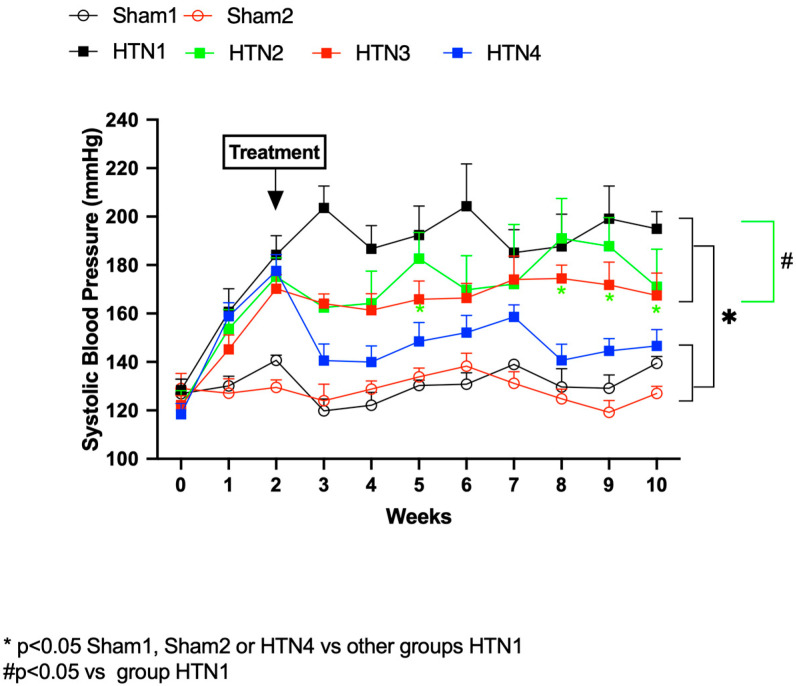
Systolic blood pressure (SBP) of normotensive and hypertensive rats. Normotensive rats were submitted to Sham surgery and treated with the carrier alone (Sham 1) or with 300 mg/kg *Kalanchoe crenata* extract (Sham 2). Hypertensive rats were submitted to the two-kidney one-clip (2K1C) procedure and treated with the carrier alone (HTN 1), with 300 mg/kg *K. crenata* extract (HTN 2), with 10 mg losartan (HTN 3), or with 300 mg/kg *K. crenata* extract and 10 mg losartan (HTN 4). Significant differences are indicated by * (*p* < 0.05) for Sham 1/Sham 2/HTN 4 versus HTN 1/HTN 2/HTN 3, and by # (*p* < 0.05) for HTN 1 versus HTN 2/HTN 3.

**Figure 5:**
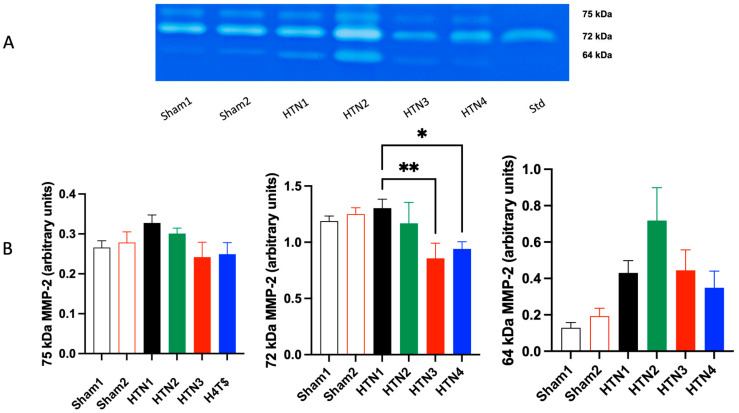
(**A**) The gel shows the zymography of the 75 kDa, 72 kDa, and 64 kDa isoforms for each experimental group together with internal standard. (**B**) Activities of MMP2 isoforms in the aortic tissue of normotensive and hypertensive rats. Normotensive rats were submitted to Sham surgery and treated with the carrier alone (Sham 1) or with 300 mg/kg *Kalanchoe crenata* extract (Sham 2). Hypertensive rats were submitted to the two-kidney one-clip (2K1C) procedure and treated with the carrier alone (HTN 1), with 300 mg/kg *K. crenata* extract (HTN 2), with 10 mg losartan (HTN 3), or with 300 mg/kg *K. crenata* extract and 10 mg losartan (HTN 4). Significant differences in relation to HTN1 are indicated by asterisks as follows: *p* < 0.05 (*) and *p* < 0.01 (**).

The SBP values recorded over the 10-week experimental period (Figure 4) demonstrated that rats treated with a combination of *K. crenata* extract and losartan (group HTN 4) presented significantly reduced SBP at the third week of administration onwards, with values that were similar to those of the Sham 1 and Sham 2 groups. Zymography of MMP2 in aortic tissues revealed that the activities of the 72 kDa isoform were reduced significantly in hypertensive animals treated with losartan (group HTN 3; *p* < 0.01) or with a combination of *K. crenata* extract and losartan (group HTN 4; *p* < 0.05) (Figure 5). In contrast, no changes in the activities of the 75 kDa and 64 kDa MMP2 isoforms were observed in any of the treatment groups.

## 3. Discussion

The initial aim of the present study was to isolate, identify, and quantify the chemical markers in the aqueous extract of *K. crenata*. Previous research involving *K. crenata* demonstrated the presence of patuletin 3-*O*-α-L-rhamnopyranosyl-7-*O*-L-rhamnopyranoside (**2**) in leaf extracts, indicating that the compound is a chemical marker for the species [27]. Considering that other studies have shown the presence of acetylated rhamnosides of patuletin in *K. brasiliensis, Kalanchoe gracilis* (a synonym of *Kalanchoe ceratophylla* Haw.), and *Kalanchoe pinnata* (Lam.) Pers. [21,28,29], we suggest that both patuletin rhamnosides isolated in the present study represent chemical markers of *K. crenata*.

Our second objective was to investigate the antihypertensive activity of the aqueous extract of *K. crenata*, administered alone or in combination with losartan. This synthetic angiotensin II receptor blocker can be used alone or with other medications to treat hypertension and left ventricular hypertrophy. There are two main animal models used to investigate the antihypertensive effects of synthetic drugs or herbal medicines, depending on the etiology of hypertension. One of them explores primary hypertension using transgenic hypertensive animals, while the other addresses secondary hypertension as, for example, renovascular hypertension induced by the 2K1C procedure [30], which we used in this study.

Our study has demonstrated that *K. crenata* extract plus losartan (HTN 4 group) improved blood pressure control in 2K1C-induced hypertensive Wistar rats. Furthermore, the observed reduction in SBP in the HTN 4 group confirmed that the interaction between the synthetic and herbal agents leads to more effective control of hypertension than either losartan or the extract alone. Hendrayana et al. [31] also demonstrated a synergistic effect between cucumber (*Cucumis sativus* L., Cucurbitaceae) fruit juice and losartan in reducing blood pressure in angiotensin II-induced hypertension in rats. In addition, Galal et al. [32] showed that administering cranberry extract in association with losartan protected rats against aluminum chloride-induced hepatorenal damage.

While the exact mechanism behind this synergistic antihypertensive effect remains to be fully elucidated, a pharmacokinetic interaction may be involved. Specifically, there may be an interaction with losartan resulting from the enzymatic inhibition of CYP3A4 or CYP2C9, which could enhance losartan’s antihypertensive effect by altering its metabolism. In fact, a previous study revealed that crude methanol and fractions of *K. crenata* exhibit a reversible, time-dependent inhibitory effect on CYP3A4 and CYP2C19 [33]. Although there is no evidence that patuletin rhamnosides inhibit these enzymes, flavonoids are well known for this effect [34,35]. While this interaction could be beneficial, it is important to exclude any potential risk of toxicity that might arise from inhibiting these enzymes. Future studies should explore this interaction in more detail.

A direct effect of patuletins can also explain, at least in part, the antihypertensive effects observed in the HTN 4 group. Patuletin has anti-inflammatory, cytotoxic, genotoxic, hepatoprotective, antiproliferative, antiplatelet, antinociceptive, and antioxidant activities [36,37]. Sadaf et al. [22] demonstrated that a methanol extract of French marigold (*Tagetes patula* L., Asteraceae), along with fractions and pure patuletin derived therefrom, showed significant blood pressure-lowering activity in a dose-dependent manner in normotensive and L-NAME-induced hypertensive rats. Administration of the nitric oxide synthase inhibitor L-NAME reduces nitric oxide bioavailability, leading to impaired endothelium-dependent vasorelaxation and changes in blood pressure and vascular reactivity. Thus, the findings of Sadaf et al. [22] suggest that the mechanism of action of patuletin is related to restoring the endothelial function.

In a previous report, Nguelefack et al. [19] stated that blood pressure and heart rate were reduced in normotensive rats treated with an *n*-butanol extract of *K. crenata*. The observed negative chronotropic effect was justified based on the potential ability of the extract to block potassium channels and, thus, delay ventricular repolarization. These findings are intriguing, given that contemporary beta-blockers are also negative chronotropic agents and are appropriate for treating heart failure since they inhibit cardiac remodeling processes [38]. However, the SBP values of normotensive animals treated with *K. crenata* extract (Sham 2 group) were similar to those of normotensive rats treated with the carrier alone (Sham 1 group). This contradictory result can be partially explained by the differential composition of the aqueous extract used in our study and the *n*-butanol extract used by the previous researchers [19].

Evaluation of the effects of the *K. crenata* extract on the activity of aortic MMP2, which constituted the third aim of our study, showed that the activity of the 72 kDA isoform of the metalloendopeptidase was reduced significantly in rats treated with losartan (HTN 3 group) or with *K. crenata* extract plus losartan (HTN 4 group), but not with *K. crenata* alone (HTN 2). Therefore, the effect was exclusively attributed to losartan since the extract alone did not affect the enzyme activity. Conversely, it is not possible to exclude that the presence of patuletin derivatives in the *K. crenata* extract contributed to the reduction of the activity of this isoform since these flavonol rhamnosides are known to possess antioxidant properties [37]. It is known that the activation of extracellular MMPs plays an important role in the development and progression of various cardiovascular diseases [39,40,41,42,43] and that oxidative stress is the main post-translational activating factor of MMP2 in the pathophysiology of systemic arterial hypertension [30,39]. Hence, compounds that possess antioxidant activity have the potential to attenuate the expression of MMP and, thereby, protect against cardiovascular remodeling [28,44]. On this basis, natural products that inhibit the harmful effects of these metalloendopeptidases are welcome in the treatment of arterial hypertension and its consequences.

Besides potentially interacting with losartan, restoring endothelial function, decreasing heart rate, and decreasing MMP2 activity, other mechanisms can be involved, such as diuretic, beta blockade, angiotensin-converting enzyme inhibition, calcium-channel blockade, or direct effects on vascular reactivity or vascular tonus, which we did not assess.

In this study, neither the *K. crenata* extract nor losartan, alone or combined, changed cardiac hypertrophy or the thickness of the middle layer of the aortic wall during the 10-week treatment period.

Regarding safety, a study involving treating mice with an aqueous extract of *K. crenata* showed that doses of 0.25 to 5 g/kg administered intraperitoneally produced no acute toxic effects [45]. However, it is important to keep in mind that substances in *K. crenata* can inhibit the CYP3A4 enzyme, which is one of the key enzymes responsible for the hepatic metabolism of numerous drugs. This should be a warning to patients who use herbal products derived from *K. crenata*, considering that they can interact with analgesics and asthma medications. Furthermore, in vitro assays have shown that an aqueous extract of the species can exert antithyroid effects, such that chronic consumption could contribute to the development of goiter and hypothyroidism, especially in individuals who present low iodine intake [46].

Our results are limited because we studied only one dose regimen and did not assess vascular reactivity or tonus. Nevertheless, this is the first study to investigate the antihypertensive effect of an aqueous extract of *K. crenata*, which is the pharmaceutical form traditionally used by folks, and the first to investigate the combined effects of *K. crenata* with losartan, a commonly used antihypertensive drug with a response rate of 63.8% to 72.1%, depending on the specific study and treatment regimen [47,48]. Finding an herbal medicine capable of increasing losartan’s response rate could benefit up to 30% of all hypertensive patients. Future studies should investigate dose regimens beyond the traditional dose used and the effect of diastolic blood pressure.

In conclusion, the aqueous extract of *K. crenata* leaves contained patuletin rhamnoside derivatives, considered the species’ chemical markers, along with the common plant metabolite phaselic acid. The extract effectively controlled SBP in 2K1C hypertensive rats when administered in combination with losartan. Additional efficacy and safety studies should be carried out to verify whether this plant species can be used as an adjuvant in treating high blood pressure and cardiovascular diseases.

## 4. Materials and Methods

### 4.1. Plant Material and Preparation of the Aqueous Extract

Permission to evaluate the bioactivities of extracts from Brazilian plants was granted by the National System of Genetic Resource Management and Associated Traditional Knowledge (SisGen-№. A8CE9A5).

Leaves of *K. crenata* were harvested in October 2022 from plants at the pre-flowering stage maintained in the gardens of the Farmácia da Natureza (Jardinópolis, SP, Brazil). Dr. José Elvino do Nascimento Júnior (Department of Natural Sciences, Federal University of São João del Rei, MG, Brazil) identified the plant material, and a voucher specimen was deposited in the Herbarium of Medicinal Plants at the University of Ribeirão Preto with the identification number HPM-2457. Fresh leaves (20.0 kg) were macerated with water (20.0 L) at room temperature (25 °C) for 2 h, after which the extract was filtered, frozen, and then lyophilized to yield 396.0 g of dry aqueous extract.

### 4.2. Isolation and Identification of the Constituents of the Aqueous Extract

A 200 g portion of the dry aqueous extract was solubilized in a methanol (100 mL): water (1 L) solution and partitioned consecutively against dichloromethane (DCM; 3 × 200 mL), ethyl acetate (EtOAc; 3 × 200 mL), and *n*-butanol (nBA; 3 × 300 mL). For each solvent, the replicate organic phases were combined and evaporated to dryness to yield 0.123 g of DCM fraction, 3.151 g of EtOAc fraction, and 8.523 g of nBA fraction. The remaining aqueous solution was freeze-dried to yield 106.7 g of residual extract.

The nBA fraction (5 g) was submitted to silica gel column chromatography (Sigma-Aldrich, St. Louis, MO, USA; 150 g; 70–230 mesh; 60 Å) with EtOAc: formic acid: water: methanol (10:0.5:0.6:0.2 *v*/*v*/*v*/*v*) as eluent to yield fractions F1 (30 mL; 1.893 g), F2 (30 mL; 0.839 g), F3 (20 mL; 0.605 g), F4 (20 mL; 0.650 g), and F5 (30 mL; 0.912 g). The entire F4 fraction was solubilized in methanol: water (2:8) and submitted to preparative HPLC on a Shimadzu (Kyoto, Japan) system comprising an LC-20AP pump, a manual injector, a DGU-20A5 online degassing unit, and a SPD-20A UV-VIS detector with CBM-20A communication bus module, and equipped with a Shimadzu ODS(L) LC-18 preparative column (50 × 25 cm; 5 µm; 100 Å). The mobile phases comprised water: formic acid (99.7:0.3 *v*/*v*) [eluent A] and methanol: acetonitrile (1:1 *v*/*v*) [eluent B] supplied at a flow rate of 20 mL min^−1^ with the following elution profile: isocratic at 5% B (0–9 min), gradient to 10% B (9.01–10 min), isocratic at 10% B (10.01–19 min), gradient to 20% B (19.01–20 min), gradient to 30% B (20.01–30 min), isocratic at 30% B (30.01–39 min), gradient to 40% B (39.01–40 min), gradient to 50% B (40.01–60 min), isocratic at 50% B (60.01–79 min), gradient to 60% B (79.01–80 min), isocratic at 60% (80.01–126 min), gradient to 100% B (126.01–170 min), and isocratic at 100% B (170.01–175 min). Detection was set at 350 nm.

The samples collected from preparative HPLC were submitted to analytical HPLC to identify, quantify, and purify the individual constituents. Analyses were performed using a Shimadzu system comprising an LC-20 AT pump, a SIL-20A autosampler, a DGU-20A3 degassing unit, and an SPD-M 20A diode array detector with a CBM-20A module, and equipped with a Phenomenex (Torrance, CA, USA) Luna C18 (250 × 4.6 mm; 5 µm; 100 Å) column. The mobile phases comprised water [eluent A] and methanol [eluent B] supplied at a flow rate of 1.0 mL/min with the following elution profile: gradient from 10 to 66% B (0–32 min), gradient from 66 to 10% (B) (32.01–35 min), and isocratic at 10% B (35.01–45 min). The injection volume was 20 µL, and detection was set at 350 nm. Sample Kc 63 (40 mg) contained a single pure component, while the semi-pure samples Kc3-80 and Kcb were further fractionated by preparative HPLC using the chromatographic system described above. The mobile phases comprised water: formic acid (99.7:0.3 *v*/*v*) [eluent A] and methanol: acetonitrile (1:1 *v*/*v*) [eluent B] supplied at a flow rate of 1.0 mL/min with the following elution profile: gradient from 10 to 100% B (0–90 min) and isocratic at 100% B (90.01–95 min). The injection volume of each sample solution (30 mg/mL) was 500 µL, and detection was set at 350 nm. The chromatographic procedure produced two pure samples labeled Kc3-83 (17 mg) and Kcb (10 mg). The three isolated components were dissolved in CD_3_OD and submitted to ^1^H, ^13^C, HSQC, HMBC, and ^1^H-^1^H COSY NMR for identification. NMR spectra were acquired on a Bruker Daltonics (Billerica, MA, USA) DRX 400 spectrometer operating at 400 MHz for ^1^H NMR and 100 MHz for ^13^C NMR, with tetramethylsilane as the internal standard.

### 4.3. Quantification of Patuletin Rhamnoside Derivatives

An accurately weighed portion (5 mg) of the dry aqueous extract was dissolved in HPLC-grade methanol (1 mL) and passed through a syringe filter (0.45 µm pore size). Samples (20 µL) were injected in triplicate onto a Sigma-Aldrich preparative reversed-phase Supelcosil™ LC-18 HPLC column (250 × 10 mm; 5 µm). The mobile phase comprised water (eluent A) and methanol (eluent B) supplied at a flow rate of 1 mL/min with a linear increase from 10 to 70% B (0–30 min).

The concentration of patuletin 3-*O*-(4″-O-acetyl-α-L-rhamnopyranosyl)-7-*O*-(3‴-*O*-acetyl-α-L-rhamnopyranoside) in the extract was determined from calibration curves constructed at concentrations of 250.0, 62.5, 15.6, 7.8, and 3.9 µg/mL in methanol (Supplementary File, Figure S1), while that of patuletin 3-*O*-α-L-rhamnopyranosyl-7-*O*-L-rhamnopyranoside was determined at concentrations of 500.0, 250.0, 125.0, 62.5, 31.25, 15.6, and 7.8 µg/mL in methanol (Supplementary File, Figure S2). The analyses were performed in triplicate with UV detection set at 330 nm.

### 4.4. Animals and Induction of Hypertension

Male Wistar rats (180–220 g) were maintained at constant temperature (22 ± 2 °C) under a 12 h light (06:00–18:00 h): 12 h dark regime and provided with chow and water *ad libitum*.

Animals were anesthetized (0.2 mL of ketamine chloride and 0.1 mL of xylazine hydrochloride applied intraperitoneally) and immediately underwent surgery to induce renovascular hypertension according to the 2K1C model described by Goldblatt et al. [49]. Unilateral renal stenosis was performed by placing a silver clip (0.2 mm i.d.) around the left renal artery, following which a single dose of oxytetracycline (0.2 g/kg) was administered intramuscularly. Sham-operated rats underwent a similar surgical procedure but without the placement of the renal artery clip. Two weeks after surgery, the animals were separated into two categories, namely hypertensive (HTN) with SBP ≥ 150 mmHg and normotensive (Sham) with SBP < 150 mmHg.

### 4.5. Treatment Groups

The dry aqueous extract of *K. crenata* was administered daily by gavage at a dose of 300 mg/kg, while losartan (Sigma-Aldrich) was administered at a dose of 10 mg/kg. Both were applied in the form of a solution in deionized water containing 0.05% Tween 80 (carrier). The dosage of the aqueous extract was based on the long experience (>30 years) of Farmácia Viva with traditional herbal medicines [50]. Furthermore, the specified dose is comparable with that (5 to 250 mg/kg) previously applied intraperitoneally in rats without showing lethal effects [45].

Six groups of animals, each comprising ten rats selected randomly from within the HTN or Sham categories, were treated via gavage daily for eight weeks. The treatment groups comprised the following: (i) Sham 1–received carrier alone; (ii) Sham 2–received *K. crenata* extract (300 mg/kg/day); (iii) HTN 1–received carrier alone; (iv) HTN 2–received *K. crenata* extract (300 mg/kg/day); (v) HTN 3–received losartan (10 mg/kg/day); and (vi) HTN 4–received *K. crenata* extract (300 mg/kg/day) in combination with losartan (10 mg/kg/day).

All animals were subjected to weekly SBP assessments performed by tail-cuff plethysmography using a Kent Scientific (Torrington, CT, USA) CODA^®^ High Throughput System. Rats were considered hypertensive when SBP was 25 mmHg higher than the basal value determined prior to surgery. The body weight of each animal was evaluated weekly throughout the study period. At the end of the 10th week after surgery, SBP was measured, and the rats were euthanized with an overdose of ketamine/xylazine anesthetic.

### 4.6. Evaluation of Organs and Tissues

The kidneys, heart, left tibia, and thoracic aorta were removed from the animals, weighed, and measured. The weights of the two kidneys were used to determine the ratio between the right (clipped) and the left (non-clipped) organ to validate the 2K1C model of hypertension. Tissue samples were fixed in 10% formaldehyde for 48 h, cut into thin sections, and stained with hematoxylin and eosin. The sections were examined using a stereoscopic magnifier and an optical microscope, and images were acquired using Leica (Wetzlar, Germany) Imaging Systems IM50 software at ×100 and analyzed using National Institutes of Health (Bethesda, MD, USA) ImageJ 1.46r software. The thickness of the middle layer of the aortic wall was measured, and cardiac samples were analyzed for ventricular thickness. Six histological measurements were performed for each animal in each treatment group. The cardiac hypertrophy index was calculated as the heart weight (g) to tibia length (cm) ratio.

### 4.7. Measurement of Aortic MMP-2 Levels

Aortic tissues were homogenized in a buffer containing 20 mmol/L Tris–HCl, pH 7.4, 1 mmol/L 1,10-phenanthroline, 1 mmol/L phenylmethylsulfonyl fluoride (PMSF), 1 mmol/L N-ethylmaleimide (NEM), and 10 mmol/L CaCl2. Tissue extracts were normalized for protein concentration, and gelatin zymography was performed as previously described (Rizzi et al., 2009, [40]). The forms of MMP-2 were identified as bands at 75, 72, and 64 kDa.

### 4.8. Statistical Analysis

Data were expressed as the mean ± standard error of the mean, while the differences between the mean values were assessed through a two-way analysis of variance (ANOVA) followed by the Bonferroni post hoc test. The *p*-values < 0.05 and <0.01 were considered significant. Adjustment for multiple comparisons across different ANOVA analyses was not done.

## Data Availability

All data relevant to the study are included in the article and its Supplementary Files. Data are available from the corresponding author upon reasonable request.

## Supplementary Materials

The following supporting information can be downloaded at: https://www.mdpi.com/article/10.3390/molecules29246010/s1.
