# Supplementary material for: Kalanchoe crenata Andrews (Haw.) Improves Losartan’s Antihypertensive Activity
Source: Molecules. 2024 Dec 20;29(24):6010. doi: 10.3390/molecules29246010 (PMC11676209; doi:10.3390/molecules29246010)
Supplement: Supplementary file 1 [file molecules-29-06010-s001.zip › molecules-3308965-supplementary.pdf]

SUPPLEMENTARY FILE

Kalanchoe crenata Andrews (Haw.) Improves Losartan's Antihypertensive Activity

Pedro de Padua G. Amatto [1], Juliana da Silva Coppede [1], Carla Renata Kitanishi [1], Giovana Graça Braga [1], Thaysa Carvalho de Faria [1], Elen Rizzi [1], Suzelei de Castro França [1], Fernanda Basso [2], Adriana Aparecida Lopes [1], Fábio Carmona [3,4], Silvia Helena Taleb Contini [1] and Ana Maria Soares Pereira [1,4,*]

[1] Department of Biotechnology of Medicinal Plants, University of Ribeirão Preto, Ribeirão Preto 14096-900, Brazil; pedro.goulart@sou.unaerp.edu.br (P.d.P.G.A.); jcoppede@unaerp.br (J.d.S.C.); ckitanishi@unaerp.br (C.R.K.); gi07braga@usp.br (G.G.B.); thaysa.tinki@hotmail.com (T.C.d.F.); elen_rizzi@yahoo.com.br (E.R.); sfranca@unaerp.br (S.d.C.F.); alopes@unaerp.br (A.A.L.); scontini@unaerp.br (S.H.T.C.)

[2] School of Dentistry, São Paulo State University Júlio de Mesquita Filho, Araraquara 14800-060, Brazil; f.basso@unesp.br

[3] Ribeirão Preto Medical School, University of São Paulo, Ribeirão Preto 14049-900, Brazil; carmona@usp.br

[4] Botanical Garden of Medicinal Plants Ordem e Progresso, Jardinopólis 14680-000, Brazil

* Correspondence: apereira@unaerp.br; Tel.: +55-16-3603-6727

Table S1 ^{1}H NMR (CD_3OD; 400 MHz) and ^{13}C NMR (CD_3OD; 100 MHz) data for compound **1** isolated from the aqueous extract of *Kalanchoe crenata* leaves: patuletin 3-*O*-(4''-*O*-acetyl-α-L-rhamnopyranosyl)-7-*O*-(3'''-*O*-acetyl-α-L-rhamnopyranoside)

**Position**	**δ_C**	**δ_H**
**2**	160.00	-
**3**	135.78	-
**4**	179.79	-
**5**	150.15	-
**6**	131.14	-
**7**	156.23	-
**8**	99.94	6.65, s
**9**	157.82	-
**10**	107.46	-
**1'**	122.71	-
**2'**	116.90	7.39, d (*J* = 2.0 Hz)
**3'**	146.71	-
**4'**	150.15	-
**5'**	116.39.	6.94, d (*J* = 8.0 Hz)
**6'**	123.02	7.33, dd (*J* = 8.0, 2.0 Hz)
**6-OCH3**	62.42	3.93, s
**3-ORha**		
**1''**	102.61	5.52, bs
**2''**	71.62	4.23, bs
**3''**	70.05	3.90, m
**4''**	74.97	4.86, m
**5''**	69.70	3.31, m
**6''**	17.61	0.80, d (*J* = 6.0 Hz)
**4''OAc (CH_3)**	21.10	2.06, s
**4''OAc (CO)**	172.67	-
**7-ORha**		
**1'''**	100.23	5.60, bs
**2'''**	69.56	4.27, bs
**3'''**	75.28	5.19, dd (*J* = 9.2, 3.2 Hz)
**4'''**	70.79	3.71, m
**5'''**	71.71	3.75, m
**6'''**	18.09	1.29, d (*J* = 6.0 Hz)
**3'''OAc (CH_3)**	21.03	2.17, s
**3'''OAc (CO)**	172.56	-

Abbreviations: d = doublet; dd = doublet of doublets; m =multiplet; s = singlet; bs = broad singlet

Table S2 ^{1}H NMR (CD_3OD; 400 MHz) and ^{13}C NMR (CD_3OD; 100 MHz) data for compound **2** isolated from the aqueous extract of *Kalanchoe crenata* leaves: patuletin 3-*O*-α-L-rhamnopyranosyl-7-*O*-L-rhamnopyranoside

Position	δ_C	δ_H
**2**	159.85	-
**3**	136.47	-
**4**	179.96	-
**5**	150.16	-
**6**	130.97	-
**7**	156.39	-
**8**	99.90	6.55, s
**9**	157.81	-
**10**	107.34	-
**1’**	122.80	-
**2’**	117.05	7.32, d (*J* = 2.0 Hz)
**3’**	146.49	-
**4’**	150.16	-
**5’**	116.57	6.84, d (*J* = 8.0 Hz)
**6’**	123.22	7.30, dd (*J* = 8.0, 2.0 Hz)
**6-OCH$_3$**	62.48	3.79, s
**3-ORha**		-
**1’’**	103.65	5.27, s
**2’’**	71.91	4.15, m
**3’’**	72.19	3.65-3.81, m
**4’’**	73.23	3.25-3.28, m
**5’’**	72.19	3.35-3.43, m
**6’’**	17.70	0.86, d (*J* = 6.0 Hz)
**7-ORha**		
**1’’’**	100.42	5.48, s
**2’’’**	71.77	4.01, s
**3’’’**	72.09	3.66-3.69, m
**4’’’**	73.54	3.35-3.43, m
**5’’’**	71.51	3.54-3.69, m
**6’’’**	18.12	1.67, d (*J* = 6.0 Hz)

Abbreviations: d = doublet; dd = doublet of doublets; m =multiplet; s = singlet

Table S3. ^{1}H NMR (CD_3OD; 400 MHz) and ^{13}C NMR (CD_3OD; 100 MHz) data of compound **3** isolated from the aqueous extract of *Kalanchoe crenata* leaves: *trans*-caffeoyl-malic acid (phaselic acid)

Position	δ_C	δ_H
**1**	172.89	-
**2**	70.05	5.47, dd (J = 8.4, 4.0 Hz)
**3a**	37.15	2.97, dd (J = 16.0, 4.0 Hz)
**3b**	37.15	2.88, dd (J = 16.0, 4.0 Hz)
**4**	173.10	-
**1'**	127.63	-
**2'**	115.17	7.06, d (J = 2.0 Hz)
**3'**	146.85	-
**4'**	149.82	-
**5'**	116.52	6.78, d (J = 8.0 Hz)
**6'**	123.23	6.96, dd (J = 8.0, 2.0 Hz)
**7'**	147.91	7.59, d (J = 15.6 Hz)
**8'**	114.23	6.31, d (J = 15.6 Hz)
**9'**	168.13	-

Abbreviations: d = doublet; dd = doublet of doublets

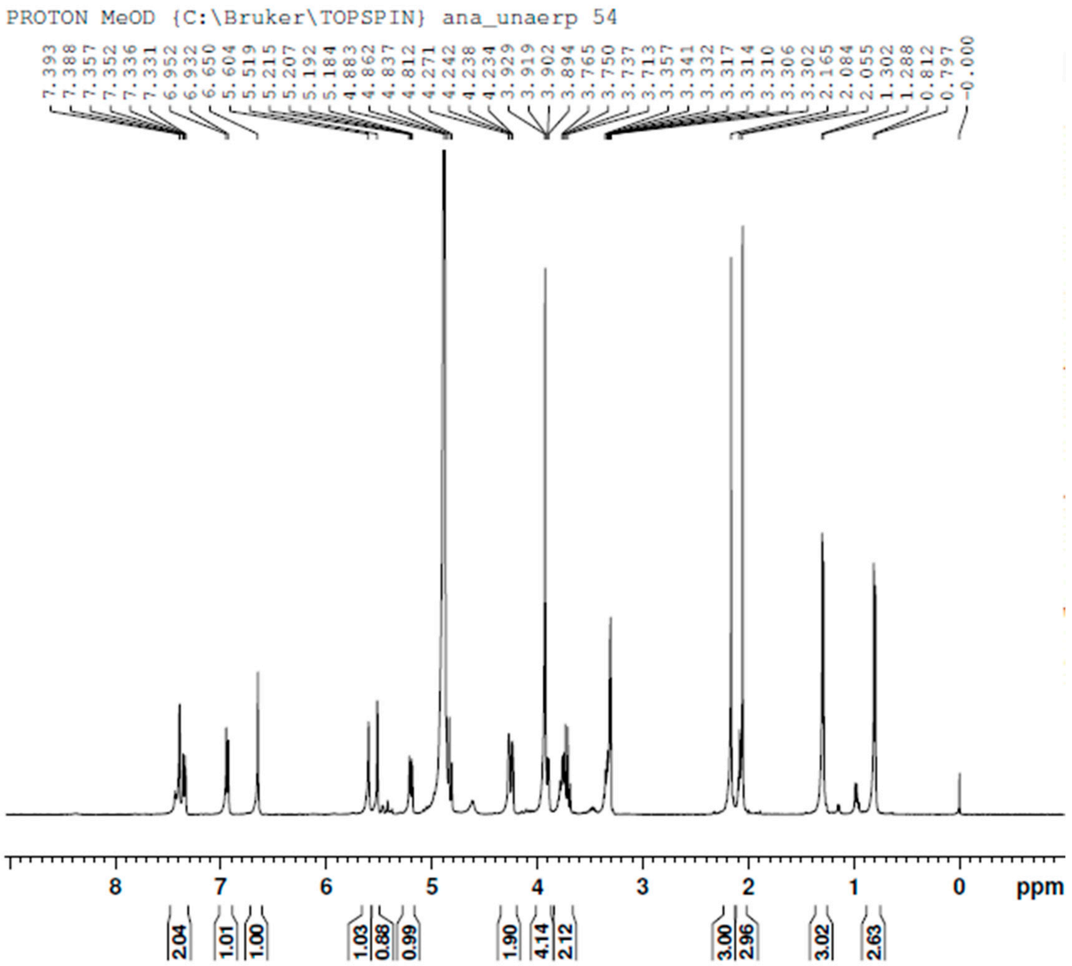

Figure S1 ^{1}H NMR (CD_3OD; 400 MHz) spectrum of compound **1** isolated from the aqueous extract of *Kalanchoe crenata* leaves: patuletin 3-*O*-(4''-*O*-acetyl-α-L-rhamnopyranosyl)-7-*O*-(3'''-*O*-acetyl-α-L-rhamnopyranoside)

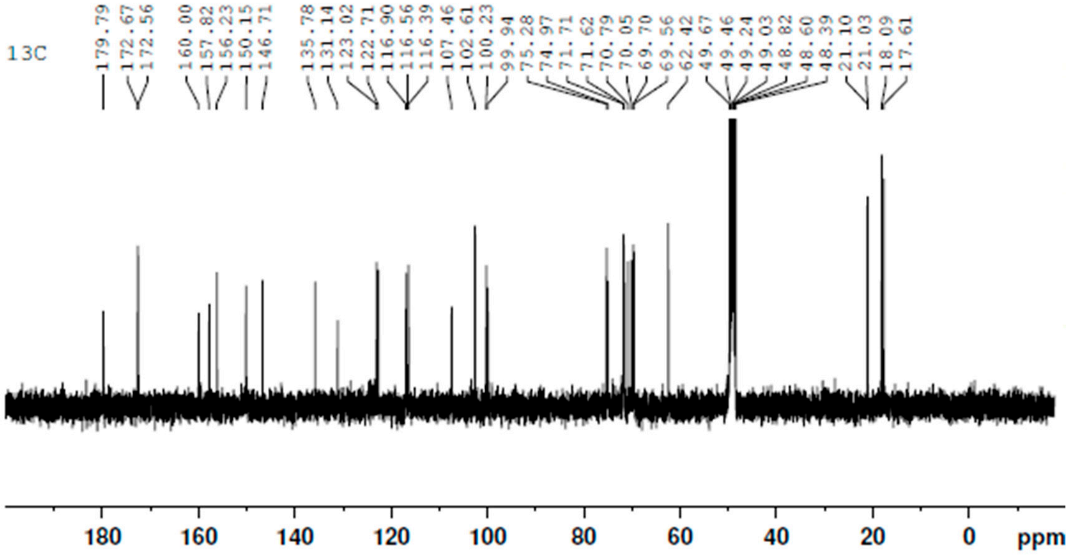

Figure S2 ^{13}C NMR (CD_3OD; 100 MHz) spectrum of compound **1** isolated from the aqueous extract of *Kalanchoe crenata* leaves: patuletin 3-*O*-(4''-*O*-acetyl-α-L-rhamnopyranosyl)-7-*O*-(3'''-*O*-acetyl-α-L-rhamnopyranoside)

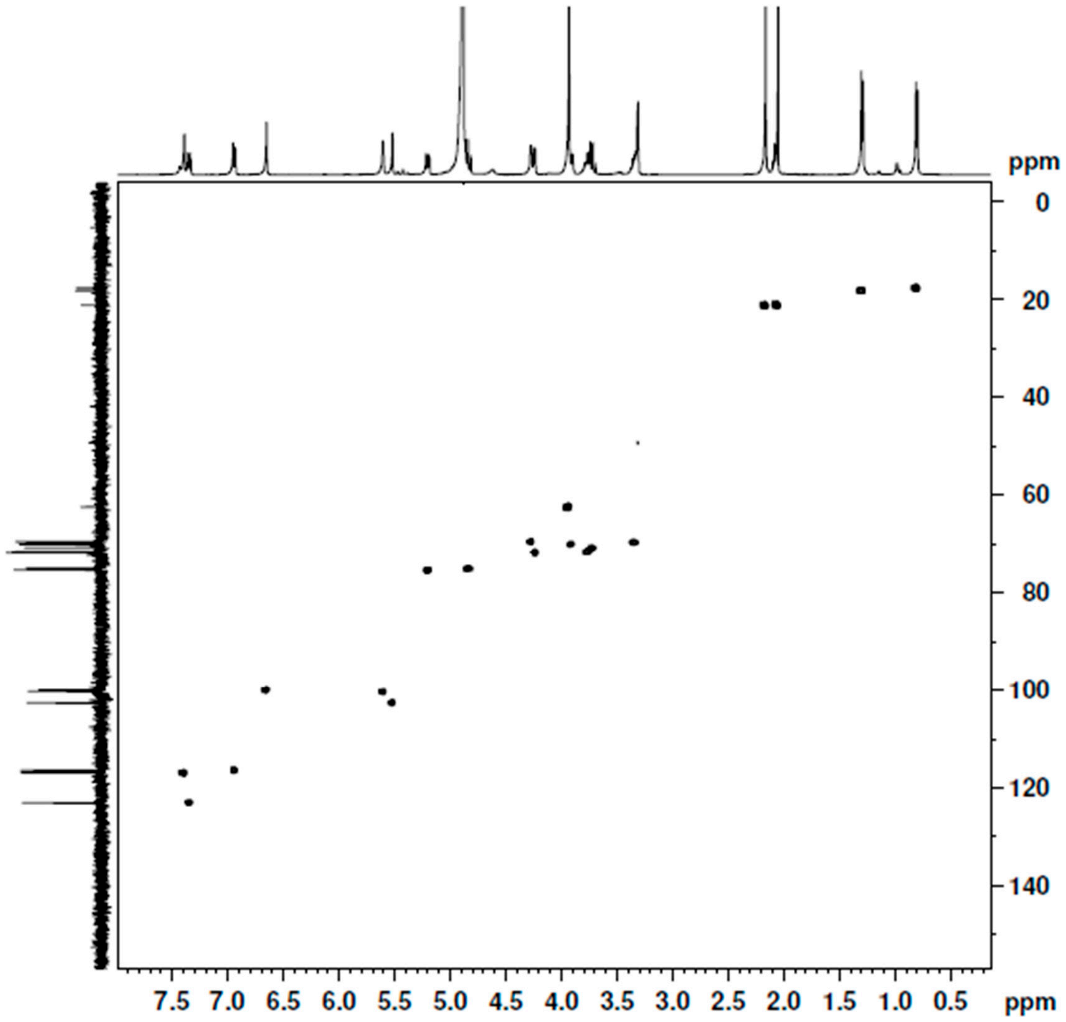

Figure S3 HSQC spectrum of compound **1** isolated from the aqueous extract of *Kalanchoe crenata* leaves: patuletin 3-*O*-(4''-*O*-acetyl-α-L-rhamnopyranosyl)-7-*O*-(3'''-*O*-acetyl-α-L-rhamnopyranoside)

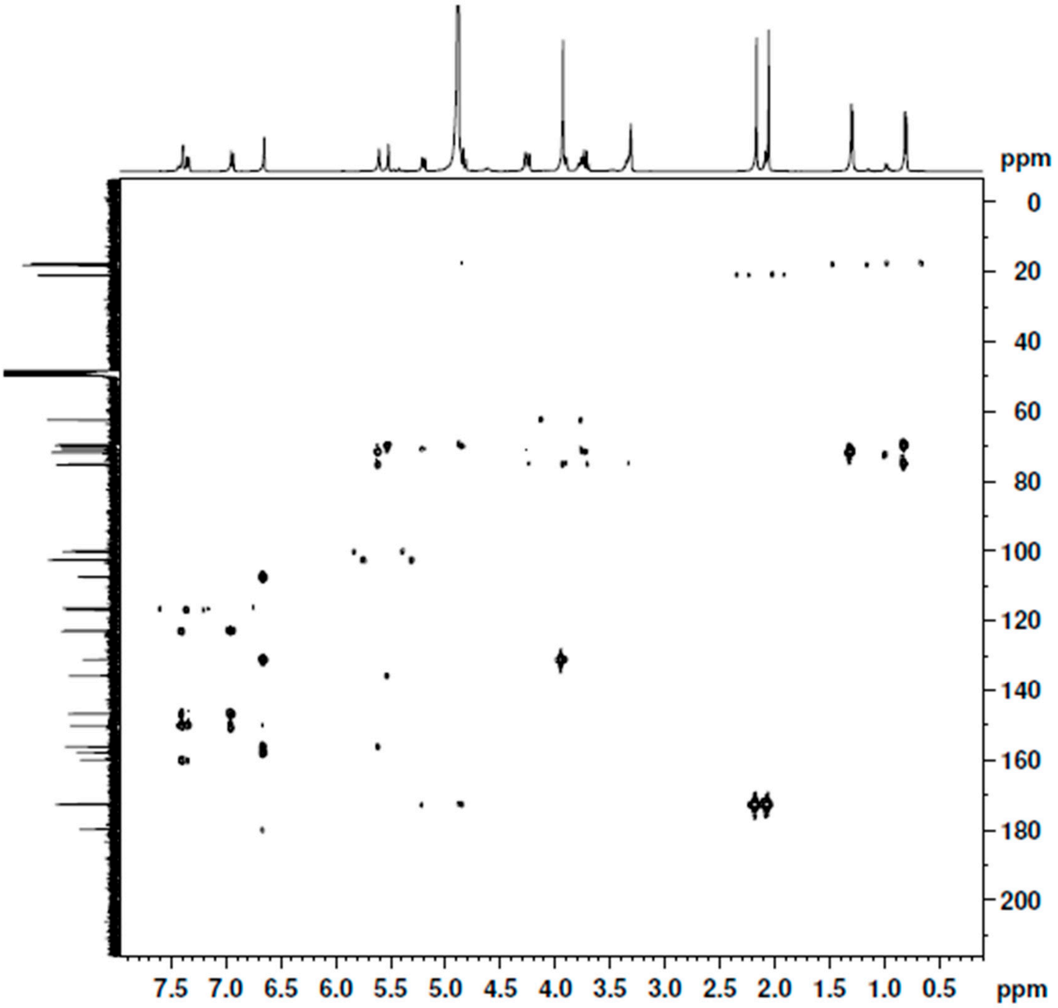

Figure S4 HMBC spectrum of compound **1** isolated from the aqueous extract of *Kalanchoe crenata* leaves: patuletin 3-*O*-(4''-*O*-acetyl-α-L-rhamnopyranosyl)-7-*O*-(3'''-*O*-acetyl-α-L-rhamnopyranoside)

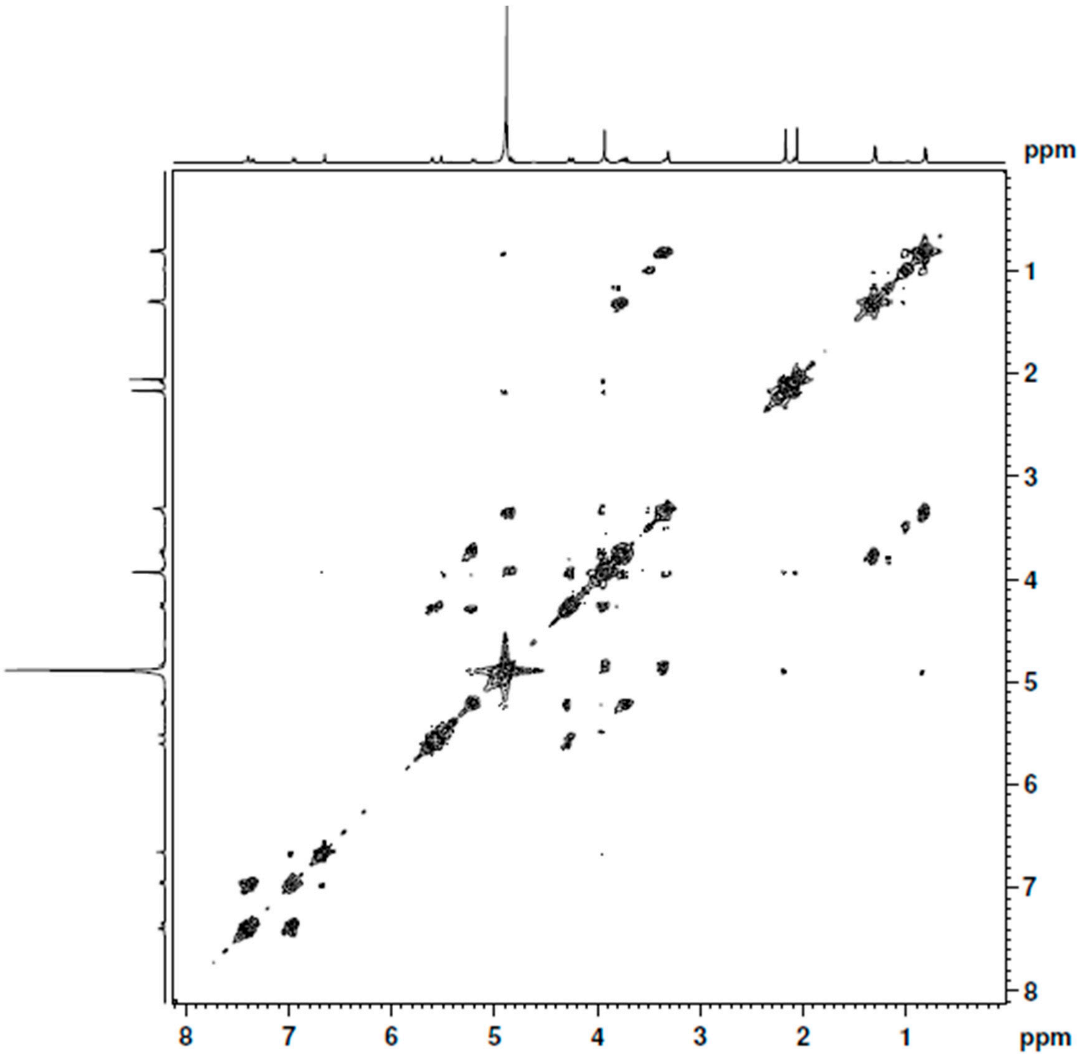

Figure S5 COSY spectrum of compound **1** isolated from the aqueous extract of *Kalanchoe crenata* leaves: patuletin 3-*O*-(4''-*O*-acetyl-α-L-rhamnopyranosyl)-7-*O*-(3'''-*O*-acetyl-α-L-rhamnopyranoside)

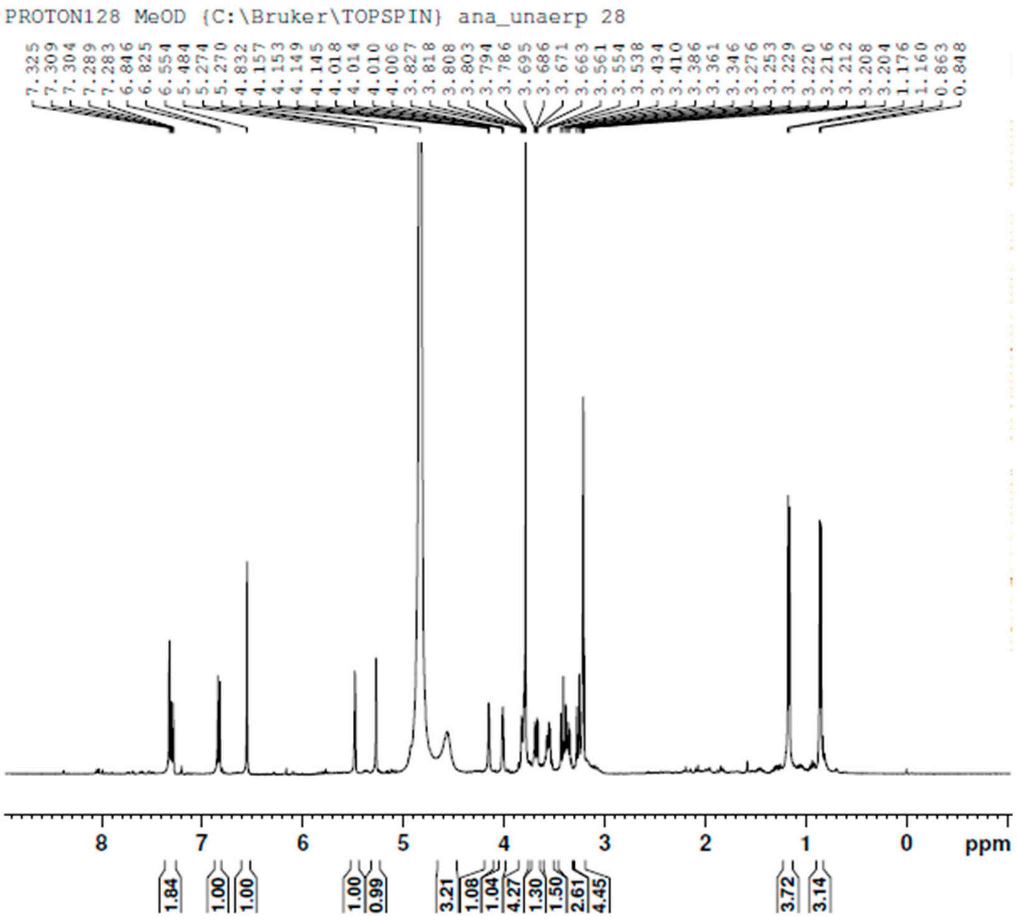

Figure S6 ^{1}H NMR (CD_3OD; 400 MHz) spectrum of compound **2** isolated from the aqueous extract of *Kalanchoe crenata* leaves: patuletin 3-*O*-α-L-rhamnopyranosyl-7-*O*-L-rhamnopyranoside

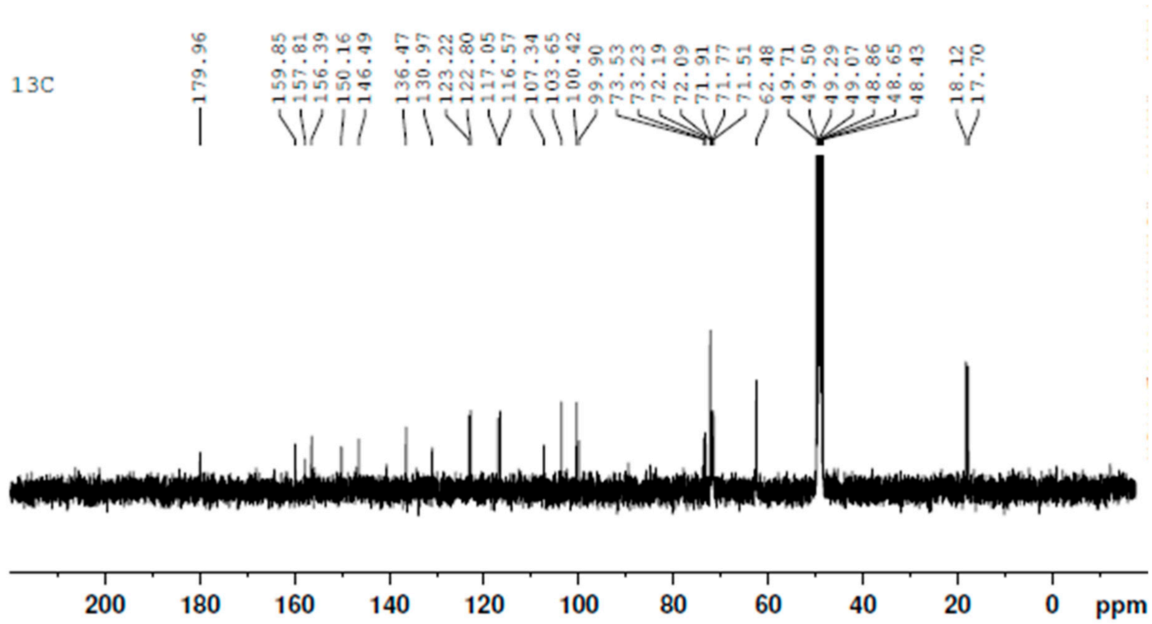

Figure S7 ^{13}C NMR (CD_3OD; 100 MHz) spectrum of compound **2** isolated from the aqueous extract of *Kalanchoe crenata* leaves: patuletin 3-*O*-α-L-rhamnopyranosyl-7-*O*-L-rhamnopyranoside

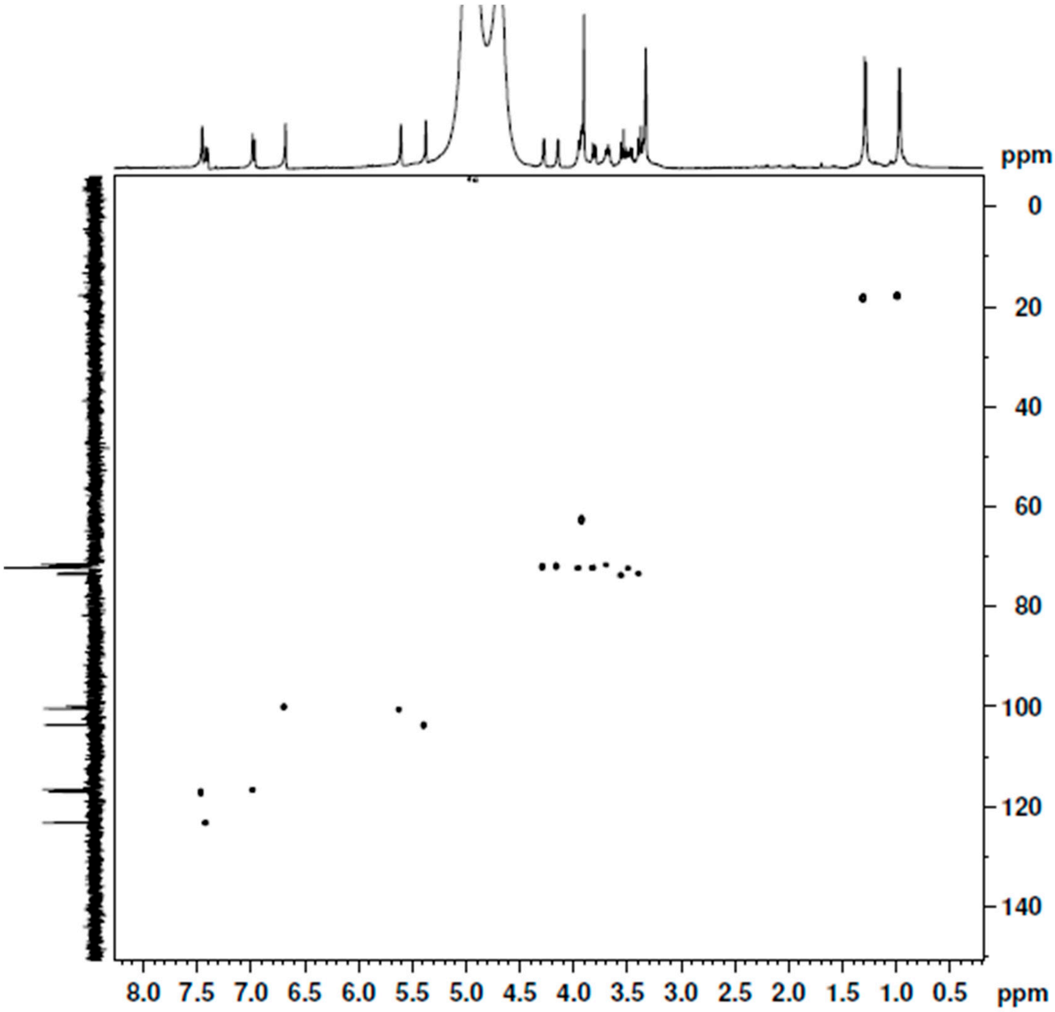

Figure S8 HSQC spectrum of compound **2** isolated from the aqueous extract of *Kalanchoe crenata* leaves: patuletin 3-O-α-L-rhamnopyranosyl-7-O-L-rhamnopyranoside

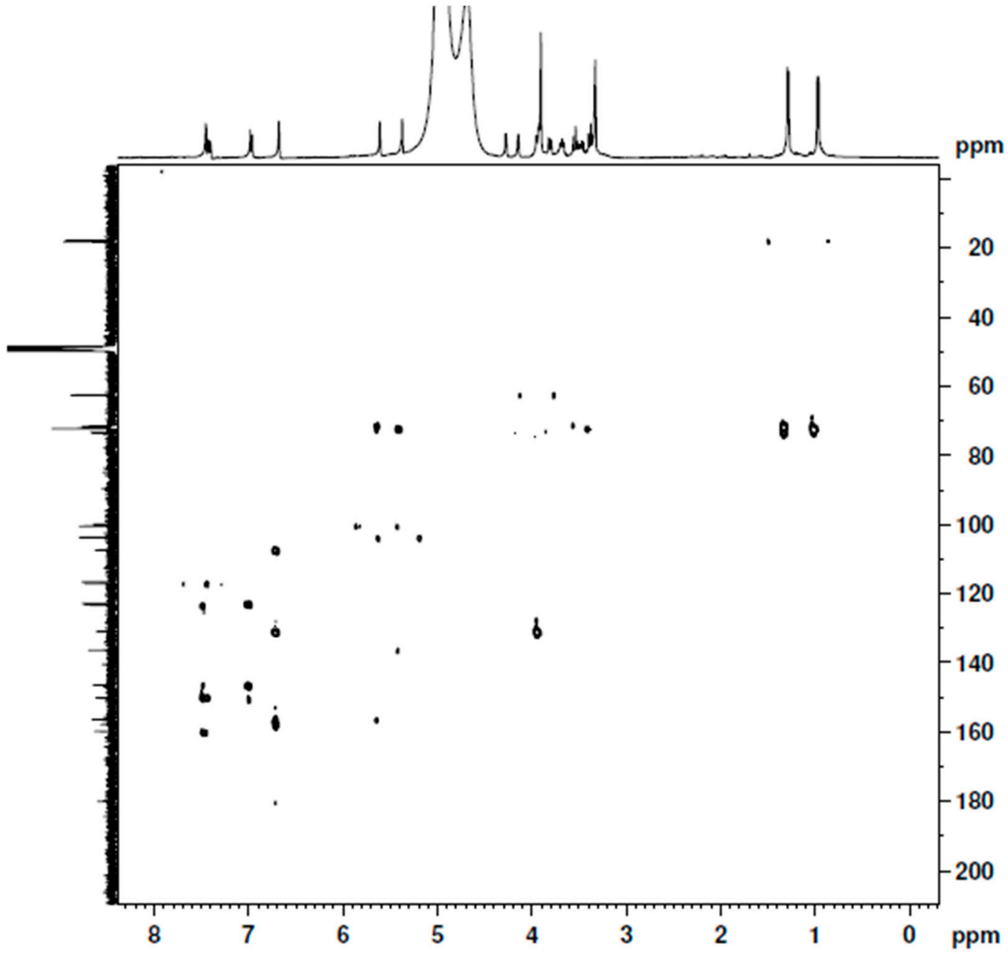

Figure S9 HMBC spectrum of compound **2** isolated from the aqueous extract of *Kalanchoe crenata* leaves: patuletin 3-*O*-α-L-rhamnopyranosyl-7-*O*-L-rhamnopyranoside

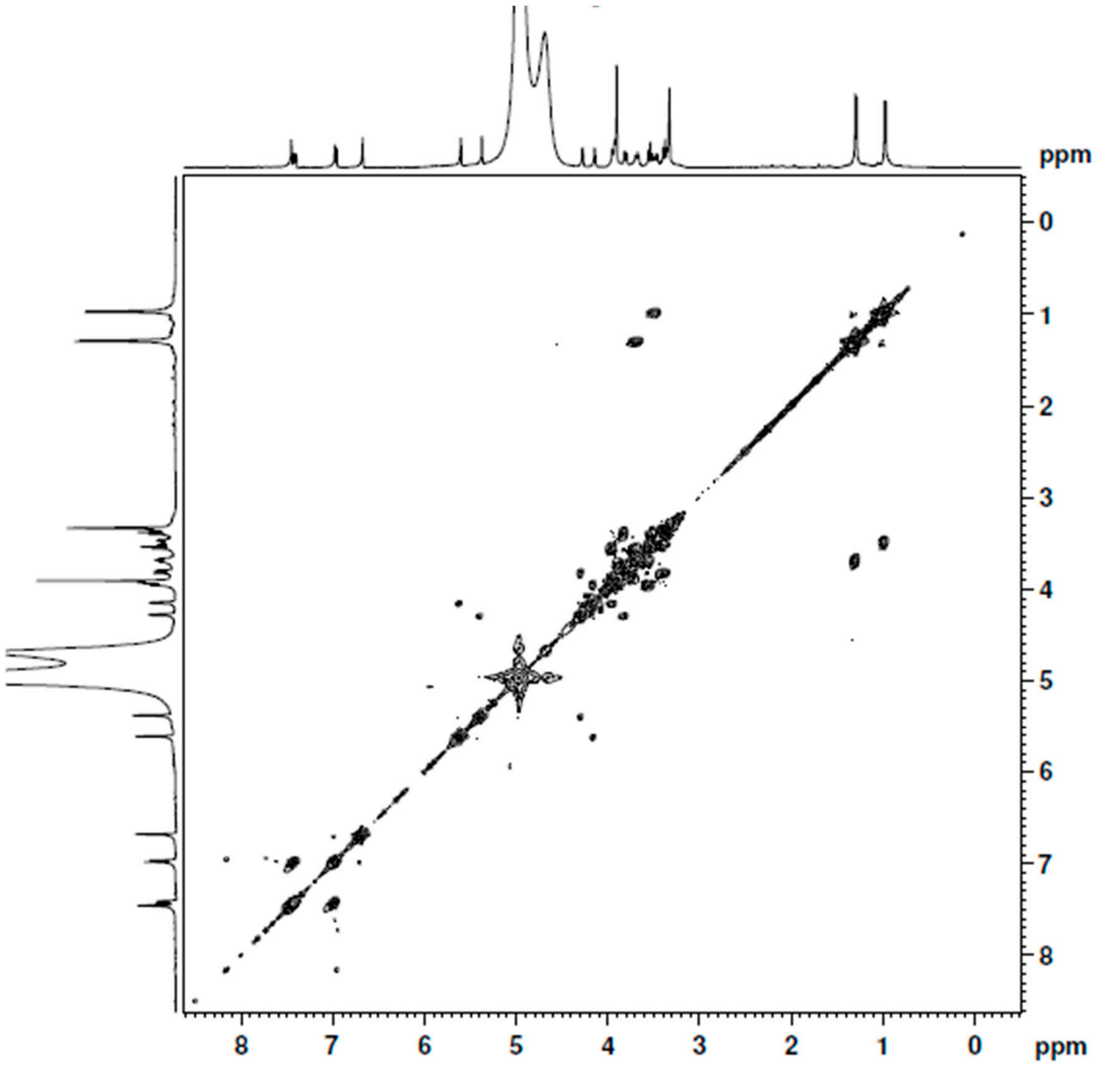

Figure S10 COSY spectrum of compound **2** isolated from the aqueous extract of *Kalanchoe crenata* leaves: patuletin 3-*O*-α-L-rhamnopyranosyl-7-*O*-L-rhamnopyranoside

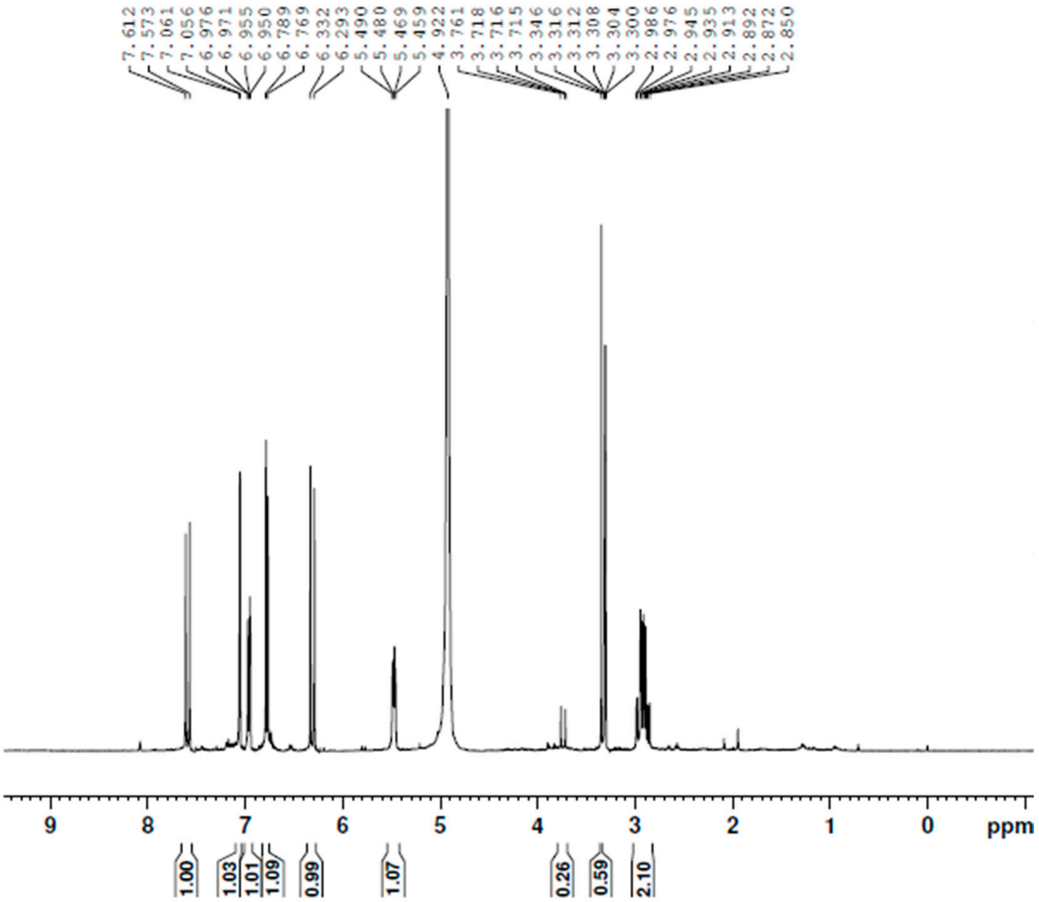

Figure S11 ^{1}H NMR (CD_3OD; 400 MHz) spectrum of compound **3** isolated from the aqueous extract of *Kalanchoe crenata* leaves: *trans*-caffeoyl-malic acid (phaselic acid)

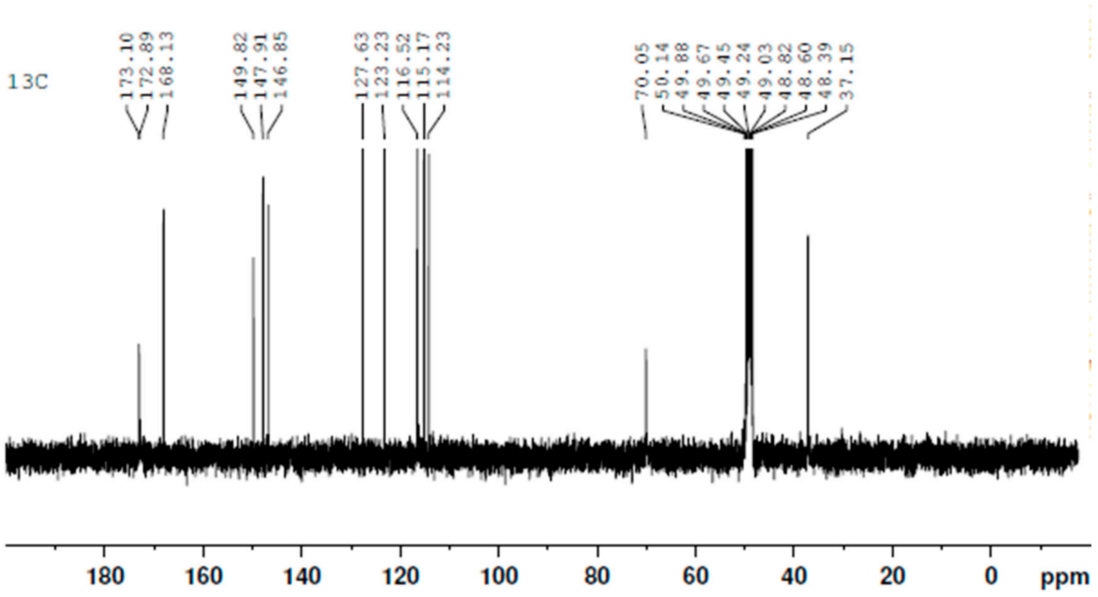

Figure S12 ^{13}C NMR (CD_3OD; 100 MHz) spectrum of compound **3** isolated from the aqueous extract of *Kalanchoe crenata* leaves: *trans*-caffeoyl-malic acid (phaselic acid)

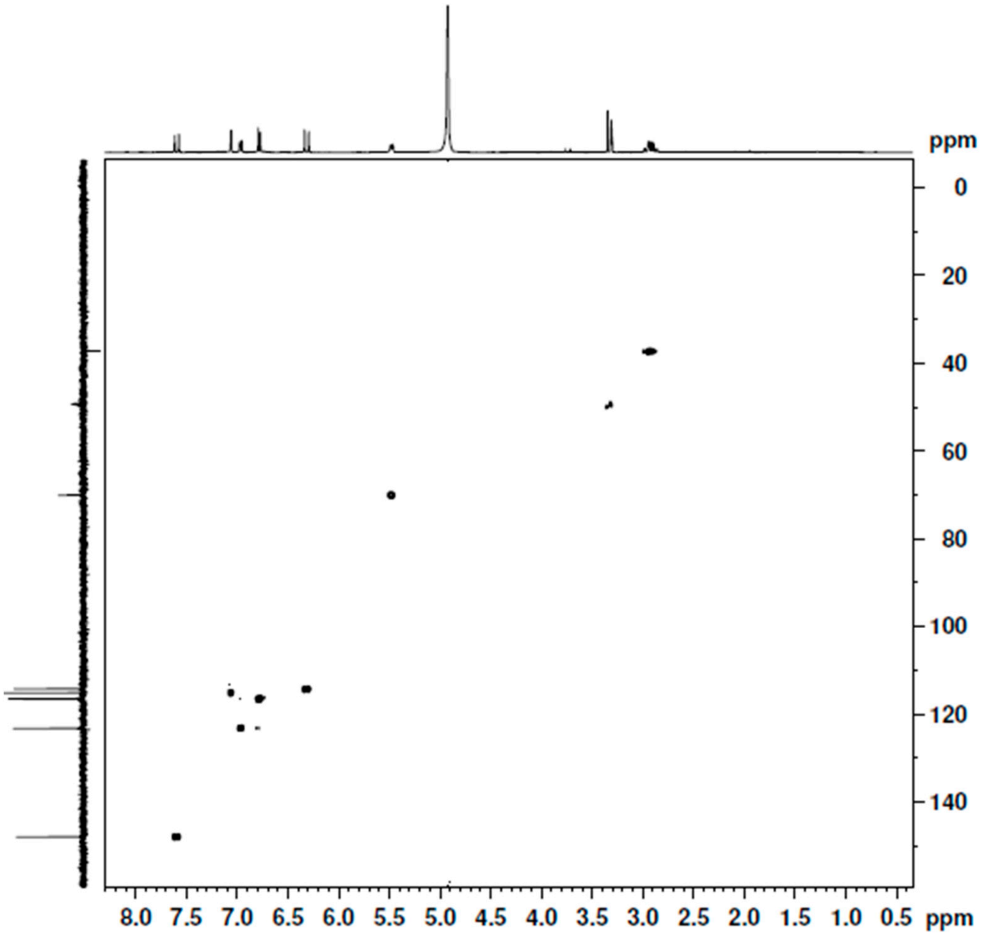

Figure S13 HSQC spectrum of compound **3** isolated from the aqueous extract of *Kalanchoe crenata* leaves: *trans*-caffeoyl-malic acid (phaselic acid)

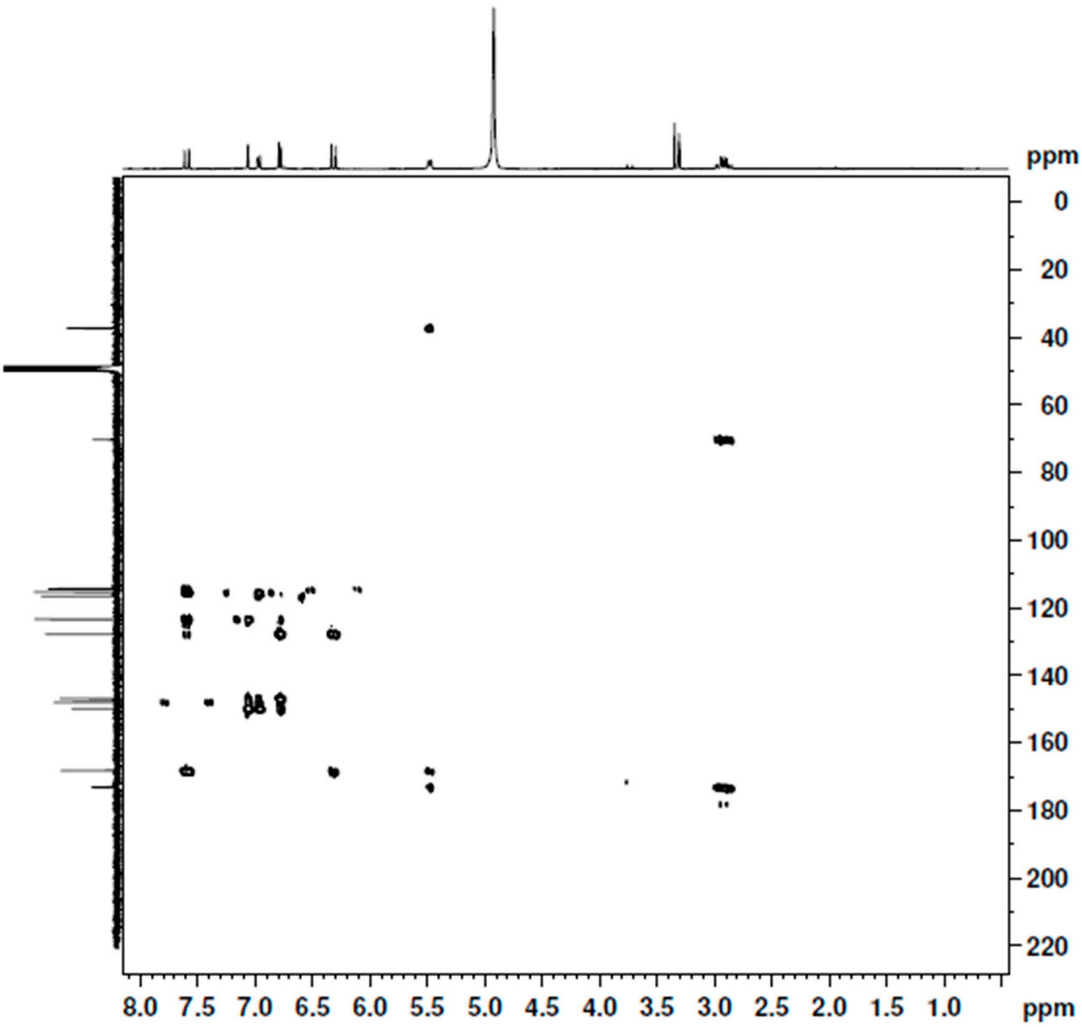

Figure S14 HMBC spectrum of compound **3** isolated from the aqueous extract of *Kalanchoe crenata* leaves: *trans*-caffeoyl-malic acid (phaselic acid)

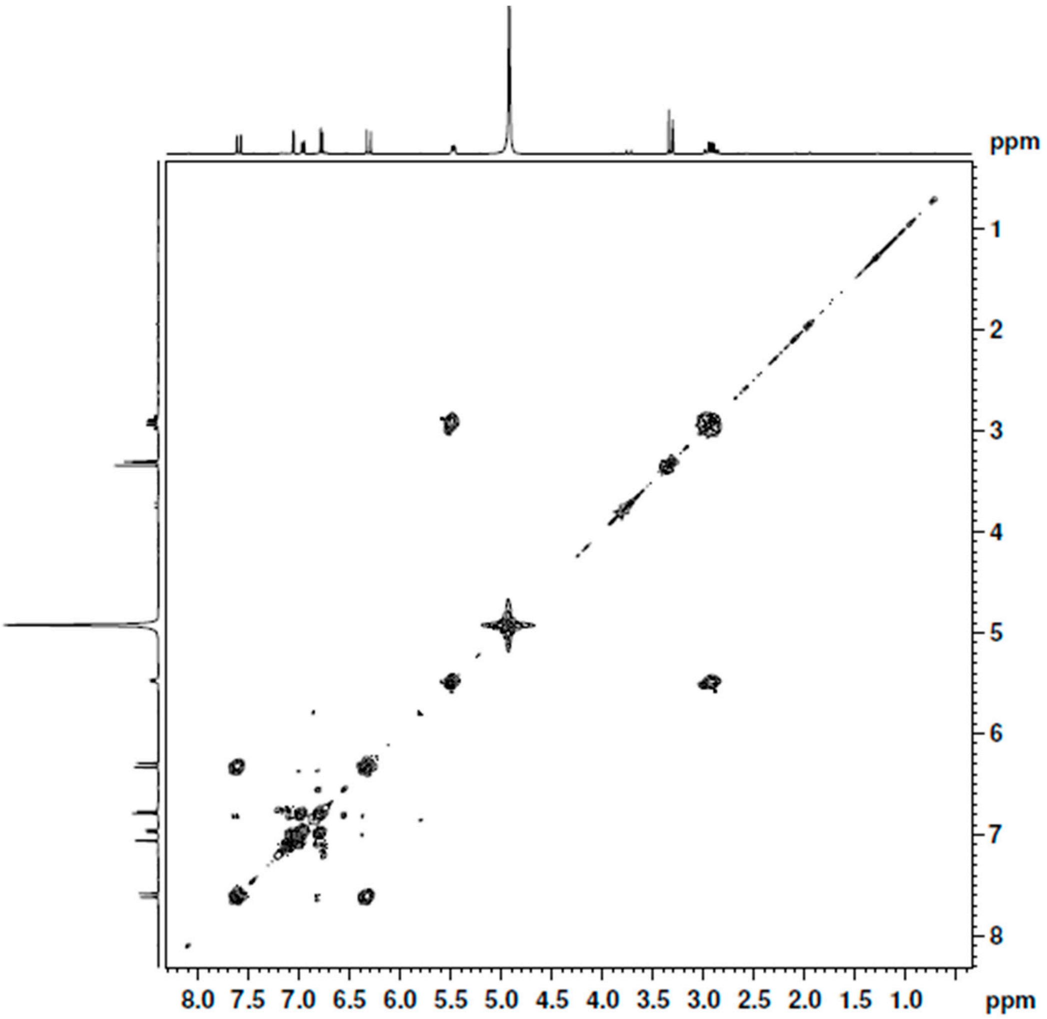

Figure S15 COSY spectrum of compound **3** isolated from the aqueous extract of *Kalanchoe crenata* leaves: *trans*-caffeoyl-malic acid (phaselic acid)

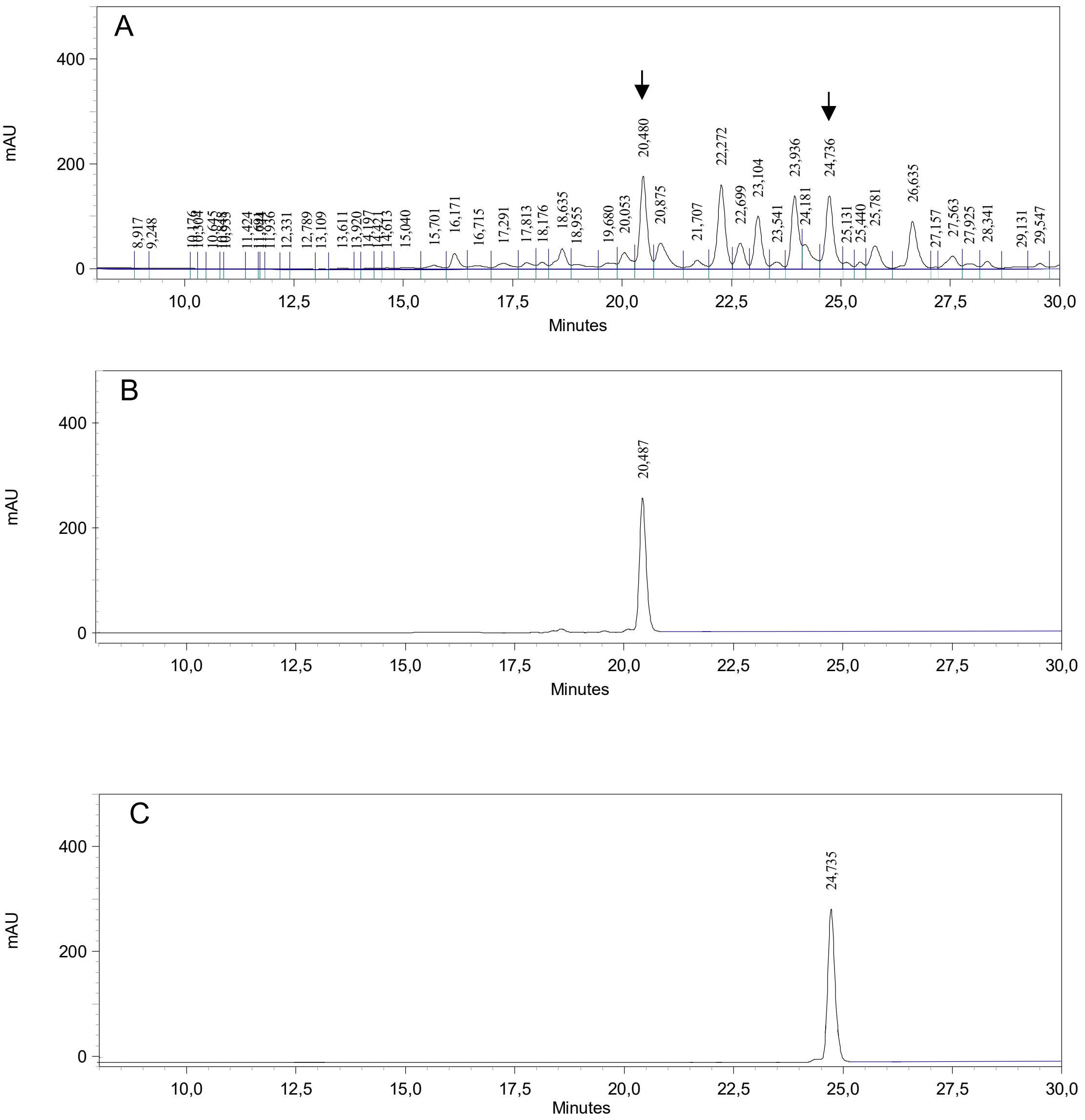

Figure S16 HPLC-DAD chromatograms of the aqueous extract of the leaves of *Kalanchoe crenata* (**A**) and the isolated compounds patuletin 3-*O*-(4''-*O*-acetyl-α-L-rhamnopyranosyl)-7-*O*-(3'''-*O*-acetyl-α-L-rhamnopyranoside) (**B**) and patuletin 3-*O*-α-L-rhamnopyranosyl-7-*O*-L-rhamnopyranoside (**C**)

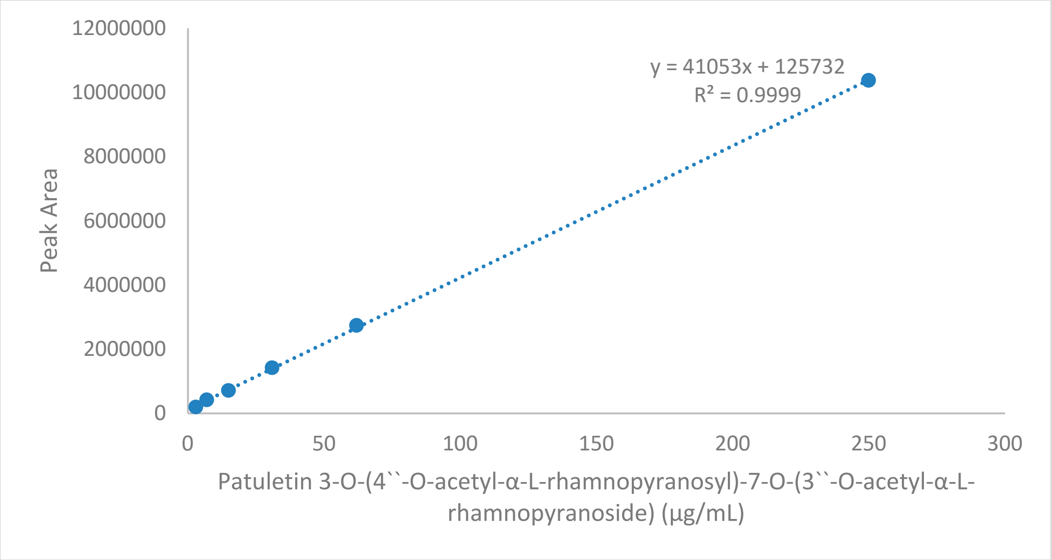

Figure S17 Calibration curve of the reference standard patuletin 3-*O*-(4''-*O*-acetyl-α-L-rhamnopyranosyl)-7-*O*-(3'''-*O*-acetyl-α-L-rhamnopyranoside). The values shown are means of triplicate determinations obtained at 330 nm

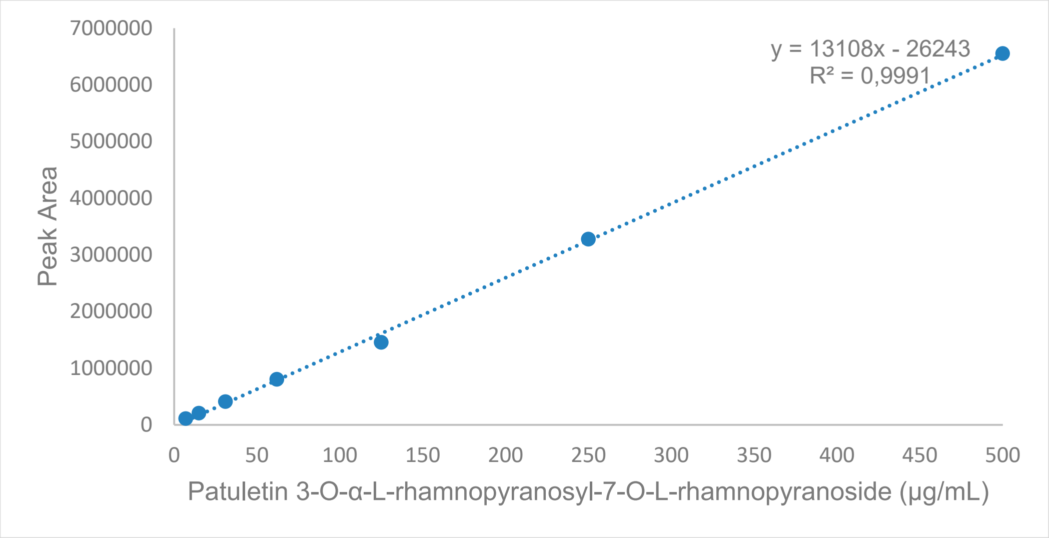

Figure S18 Calibration curve of reference standard patuletin 3-*O*- α-L-rhamnopyranosyl-7-*O*-L-rhamnopyranoside. The values shown are means of triplicate determinations obtained at 330 nm.
